# Functional specialization of chloroplast vesiculation (*CV*) duplicated genes from soybean shows partial overlapping roles during stress-induced or natural senescence

**DOI:** 10.3389/fpls.2023.1184020

**Published:** 2023-06-06

**Authors:** Andrea Luciana Fleitas, Alexandra Castro, Eduardo Blumwald, Sabina Vidal

**Affiliations:** ^1^ Laboratorio de Biología Molecular Vegetal, Instituto de Química Biológica, Facultad de Ciencias, Universidad de la República, Montevideo, Uruguay; ^2^ Department of Plant Sciences, University of California, Davis, Davis, CA, United States

**Keywords:** soybean, drought, chloroplast vesiculation, senescence, slow wilting

## Abstract

Soybean is a globally important legume crop which is highly sensitive to drought. The identification of genes of particular relevance for drought responses provides an important basis to improve tolerance to environmental stress. Chloroplast Vesiculation (CV) genes have been characterized in Arabidopsis and rice as proteins participating in a specific chloroplast-degradation vesicular pathway (CVV) during natural or stress-induced leaf senescence. Soybean genome contains two paralogous genes encoding highly similar CV proteins, *CV1* and *CV2*. In this study, we found that expression of *CV1* was differentially upregulated by drought stress in soybean contrasting genotypes exhibiting slow-wilting (tolerant) or fast-wilting (sensitive) phenotypes. *CV1* reached higher induction levels in fast-wilting plants, suggesting a negative correlation between *CV1* gene expression and drought tolerance. In contrast, autophagy (ATG8) and ATI-PS (ATI1) genes were induced to higher levels in slow-wilting plants, supporting a pro-survival role for these genes in soybean drought tolerance responses. The biological function of soybean CVs in chloroplast degradation was confirmed by analyzing the effect of conditional overexpression of CV2-FLAG fusions on the accumulation of specific chloroplast proteins. Functional specificity of *CV1* and *CV2* genes was assessed by analyzing their specific promoter activities in transgenic Arabidopsis expressing GUS reporter gene driven by *CV1* or *CV2* promoters. *CV1* promoter responded primarily to abiotic stimuli (hyperosmolarity, salinity and oxidative stress), while the promoter of *CV2* was predominantly active during natural senescence. Both promoters were highly responsive to auxin but only *CV1* responded to other stress-related hormones, such as ABA, salicylic acid and methyl jasmonate. Moreover, the dark-induced expression of *CV2*, but not of *CV1*, was strongly inhibited by cytokinin, indicating similarities in the regulation of *CV2* to the reported expression of Arabidopsis and rice CV genes. Finally, we report the expression of both *CV1* and *CV2* genes in roots of soybean and transgenic Arabidopsis, suggesting a role for the encoded proteins in root plastids. Together, the results indicate differential roles for *CV1* and *CV2* in development and in responses to environmental stress, and point to *CV1* as a potential target for gene editing to improve crop performance under stress without compromising natural development.

## Introduction

Soybean (*Glycine max* (L.) Merr.) is the most economically important oilseed crop produced worldwide and the largest source of vegetable oil and protein for human and animal consumption. Most of the soybean production is carried out in the American continent (USDA, 2022), where is cultivated during summer, mostly under rainfed agricultural practices (Cattivelli et al., 2008). Under these conditions, the occurrence of seasonal droughts unavoidably affects soybean productivity which can be reduced by up to 40% (Cattivelli et al., 2008; Le et al., 2012). Soybean is considered the most drought-sensitive plant among legume species (Clement et al., 2008). Recent studies have shown that global agricultural productivity has exhibited substantial variations during the past decades, and that these variations can be explained by changes in the drought index (Leng and Hall, 2019). In these studies, soybean was the crop that showed the highest correlation between yield reduction and drought conditions, which highlights the importance of increasing drought tolerance in soybean breeding programs.

Breeding soybean for the development of drought tolerant high yielding cultivars has posed major challenges. One of the first symptoms of drought stress in soybean is canopy wilting, which is caused by the loss of cell turgor pressure as a result of cellular dehydration (Carter, 1999; King et al., 2009). Several soybean plant introductions (PIs) have been reported during the last decades to exhibit slow canopy wilting phenotype under water deficit conditions (Carter, 1999; Kunert and Vorster, 2020). Since then, this trait has been extensively used as a visual marker to select and develop drought-tolerant, soybean genotypes in the field (Sloane et al., 1990; Charlson et al., 2009; Kunert and Vorster, 2020). In addition, there is significant evidence supporting that the slow canopy wilting phenotype in soybean is linked to drought tolerance without affecting yield (Sloane et al., 1990; Kunert and Vorster, 2020).

Slow wilting has been shown to be associated with different water-conservation mechanisms, including improved water use efficiency (WUE) (Charlson et al., 2009; Abdel-Haleem et al., 2012; Chamarthi et al., 2021), canopy temperature (Bazzer and Purcell, 2020), and better root exploratory capacity (Pantalone et al., 1996). Despite the fact that the precise molecular mechanisms underlying slow wilting phenotype are not fully understood, it is agreed that this trait is a multigenic, moderately inheritable characteristic, which benefits crop performance under drought conditions without significantly affecting yield (Ries et al., 2012; Prince et al., 2015; Kunert and Vorster, 2020).

Crop productivity is affected by the amount of light intercepted by the leaves and biomass accumulation through photosynthesis. Drought affects both biomass accumulation and plant growth, striking photosynthesis and transpiration (Tardieu et al., 2018). Drought can also trigger premature senescence, which compromises biomass accumulation in cultivated species (Reguera et al., 2013). Stress-induced senescence is the main cause of losses in biomass and in grain filling (Pourtau et al., 2006; Aoyama et al., 2014), and is also responsible for the short post-harvest life of many vegetables, fruits and flowers (Reguera et al., 2013).

Senescence is a developmental regulated process that involves various molecular and physiological events controlled at the genetic level and modified by environmental conditions (Balazadeh et al., 2008). This process can also be artificially induced by darkness (dark-induced senescence), sugars, or by hormone treatments (Balazadeh et al., 2008; Sobieszczuk-Nowicka et al., 2018). During senescence, plants recycle nutrients from their source organs to their reproductive organs (Gepstein and Glick, 2013). Thus, in grain crops, senescence highly affects plant productivity as it is linked to the production of mature seeds.

Manipulating leaf senescence through breeding or genetic engineering has been proposed as a strategy to improve tolerance to environmental stress (Rivero et al., 2007; Reguera et al., 2013; Sade et al., 2018). The stay-green phenotype, found in genotypes of different plant species including soybean, is characterized by an apparent or actual delay in leaf senescence, which in many cases, occurs as a consequence of the failure to degrade chlorophyll, chlorophyll binding proteins and/or Rubisco (Guiamét and Gianibelli, 1994; Hörtensteiner, 2009). In general, the stay-green trait enables plants to maintain and prolong the photosynthetic activity of leaves after anthesis and is often associated with increased grain yield under stress conditions (Gregersen et al., 2013). A number of studies support the use of this trait to improve crop productivity and stress tolerance in cereals by delaying leaf senescence and modulating nitrogen (N) and carbon (C) dynamics (Gregersen et al., 2013; Kamal et al., 2019). Nevertheless, soybean natural stay-green genotypes exhibit a normal timing of senescence initiation and thus, the mutations present in these genotypes are considered as non-functional or merely cosmetic (Kamal et al., 2019). Moreover, soybean genotypes carrying these types of mutations have been shown to be more susceptible to water deficit than the wild type (Luquez and Guiamét, 2002).

An alternative and successful approach to delay leaf senescence and improve stress tolerance has been carried out in transgenic tobacco plants overexpressing isopentenyltransferase (IPT), a key enzyme for cytokinin (CK) biosynthesis, under the control of a senescence-inducible promoter. Under water deprivation conditions, transgenic plants exhibited a functional stay-green phenotype and mounted a powerful acclimation response, with stimulation of the photosynthetic rate, greater WUE and minimal yield losses (Rivero et al., 2007). The stimulation of CK production has also been shown to improve ROS scavenging and promote the maintenance of photosynthetic capacity (Rivero et al., 2010; Ha et al., 2015) by increasing chloroplast stability under stress conditions (Rivero et al., 2009).

During leaf senescence, chloroplasts, which contain 75% of the N in the leaf (50% of which is supplied by RubisCO; Diaz et al, 2005), are the first organelles to be degraded. Meanwhile the nucleus and mitochondria remain active until the culmination of cell death to allow the expression of genes and the production of metabolic energy that enable the progression of senescence (Buchanan-Wollaston et al., 2005).

Chloroplast degradation involves the well-orchestrated degradation and remobilization of chlorophyll, protein and lipids. The thylakoid membranes are a very abundant source of C that is remobilized. N appears mainly in the form of proteins, so proteolysis steps are essential for remobilization, which is especially important during the reproductive stage (James et al., 2019).

Several vesicular pathways involved in bulk degradation of chloroplast components are known to operate in plants. These pathways can be dependent or independent of autophagy-related genes (ATG) (Domínguez and Cejudo, 2021). ATG-dependent pathways involve RubisCO containing bodies (RCB) and ATG8-interacting protein 1-plastid associated bodies (ATI-PS). In contrast to RCB, ATI-PS does not require a functional autophagic machinery, but functional autophagy is required for internalization in the central vacuole (Michaeli et al., 2014). While RCB transports chloroplast stroma proteins, ATI1 interacts with various chloroplast proteins, many of which are located in thylakoids and are involved in photosynthesis, such as LHCA4, PsbS, PrxA, FNR1 and APE1 (Michaeli et al., 2014; Buet et al., 2019; Zhuang and Jiang, 2019). ATG-dependent pathways allow nutrient remobilization under energy-limiting conditions and/or removal of damaged chloroplast components upon different stress conditions (Michaeli et al, 2014; Ishida et al., 2014; Dominguez and Cejudo, 2021; Xie et al., 2015; Masclaux-Daubresse et al., 2017; Chiba et al., 2003; Izumi and Ishida, 2011). Interestingly, several mutants in autophagy genes exhibit a phenotype of accelerated senescence and higher sensitivity to abiotic stress (Buet et al., 2019; Jiang et al., 2020), indicating an important role for these pathways in plant development and adaptation to extreme environmental conditions.

ATG-independent pathways involve the senescence associated vacuoles (SAVs) and the chloroplast vesiculation containing vesicles (CVV) (Guo et al., 2021). SAVs are not just transport vesicles as they display high proteolytic activity which turns them into lytic compartments themselves. The cysteine protease SAG12 (senescence-associated gene 12) is among the proteases that accumulate in these compartments (Otegui et al., 2005; Buet et al., 2019). Like RCBs, SAVs contain chloroplast proteins such as RubisCO and GS2 (Martínez et al., 2008). In addition, some thylakoid proteins, such as PsaA and Lhcas, have been detected in SAVs. However, no PSII proteins, such as D1 or PSII antenna proteins (LHCII), have been found in SAVs (Martínez et al., 2008; Gomez et al., 2019). Recently, it was shown that SAVs also participate in chlorophyll degradation during senescence (Gomez et al., 2019).

CVV is the most recently described chloroplast degradation pathway, that like SAVs, is independent of ATG. It involves a chloroplast-targeted protein named CV which participates in both natural and stress-induced senescence (Wang and Blumwald, 2014). CV interacts mainly with thylakoid proteins causing the formation of CV-containing vesicles which carry chloroplast proteins into the central vacuole where they are degraded (Wang and Blumwald, 2014). Arabidopsis *CV* gene is upregulated in response to abiotic stress and is negatively regulated by CK (Wang and Blumwald, 2014). Constitutive *CV* overexpression is lethal. Nonetheless, inducible *CV* overexpression promotes the formation of CVVs and accelerates senescence, affecting the content of photosystem proteins (PsaB, PsaA and PsbO), and stroma proteins (GS2). Furthermore, downregulation of *CV* genes promotes chloroplast stability, delays stress-induced senescence and enhances abiotic stress tolerance (Wang and Blumwald, 2014; Sade et al., 2018; Ahouvi et al., 2022).

We have previously analyzed the physiological and molecular responses of two soybean genotypes showing contrasting phenotypes under water deficit conditions; the drought sensitive TJS2049 cultivar, and the slow wilting N7001 cultivar, exhibiting stable productivity under a variety of environmental conditions (Gallino et al., 2018). Under dehydration conditions, N7001 exhibited higher WUE and accumulated more dry matter than TJS2049, supporting that the slow wilting genotype was more tolerant to drought stress. Using cDNA subtracted libraries enriched in drought-induced genes from N7001 or from TJS2049, we identified a number of differentially expressed genes involved in vesicular pathways, including the autophagy marker ATG8, the senescence associated protease SAG12 and CV encoding genes.

Given the significant evidence supporting that slow-wilting phenotype involves mechanisms that prevent yield reduction under stress (Sloane et al., 1990; Kunert and Vorster, 2020), and that stress-induced premature senescence has a major impact on crop yield, in this work we analyzed the contribution of different vesicular pathways for protein degradation in the response to water deficit of slow wilting N7001 genotype, and compared the responses to those exhibited by TJS2049.

We showed that the soybean CVV pathway is activated by water deficit, primarily in the drought sensitive genotype. Moreover, we showed that soybean *CV1* and *CV2* gene paralogous differentially contribute to abiotic stress responses and developmental cues through differences in their regulatory regions and gene expression. While previously characterized *CV* genes are involved in both stress-induced and natural senescence, in soybean these functions seem to be partitioned in two separate genes with only partially overlapping expression profiles. These results support the use of soybean single *CV* genes as potential targets suitable for gene editing as a strategy for increasing stress tolerance without affecting development.

## Materials and methods

### Plant material and growth conditions

The soybean genotypes used were N7001 (Carter et al., 2003) and TJS2049 (Pardo et al., 2014). Soybean plants were grown in a growth chamber in sand/vermiculite (1/1 ratio), with a 16/8 h (light/darkness) photoperiod, with a photon flux of 800 µmol m^-2^ s^-1^ using metallic halogen lamps (400 W) and sodium incandescent lamps (75 W) and temperatures of 30 and 20°C for day and night, respectively. Plants were watered daily to field capacity, with Rigaud and Puppo (1975) medium supplemented with 10 mM of KNO_3_. In order to reach maximum water retention capacity of the substrate, pots were watered up until excess water drained from the bottom of the pots through the metal mesh. The pots were kept in this condition for 24 h, until no water excess drainage was observed. Maximum volume of water held by the substrate was quantified and the resulting value was used as reference to express the percentage of water retained by the soil substrate during drought stress.

*Arabidopsis thaliana* accession Columbia 0 (alias Col-0) was used in this study. Seeds were surface sterilized with a solution containing 7% of bleach and 0.05% Tween-20, rinsed several times with sterile distilled water and incubated at 4°C for 3 days for stratification before placing them in Petri dishes. Plants were grown *in vitro* in half strength MS medium (2.4 g/L Murashige and Skoog, 0.5 g/L 2-(N-morpholino) ethanesulfonic acid hydrate and 0.5% phyto agar, pH 5.7), at 22°C with a 16/8 h day/night cycle and a photon flux of 120 µmol photons m^-2^ s^-1^.

Arabidopsis plants were also grown in pots filled with a mixture of moss peat and vermiculite (1:1). Seeds were germinated in sterile conditions on MS plates, transferred to pots (covered with a plastic dome during 2 days), and allowed to grow at 22°C, under a 16/8 h (light/darkness) regime, using half strength MS for irrigation.

### Soybean experimental conditions

Soybean plants were grown at maximum substrate water retention capacity until they reached the V2 (vegetative 2) developmental stage (plants with two sets of unfolded trifoliolate leaves).

Dehydration conditions were imposed by withholding irrigation, until substrate water capacity reached 25% of maximum retention capacity (water potential Ψ = ~ 2.2 MPa). Non-stressed control plants, at V2 stage, continued to be daily irrigated at maximum substrate water capacity (100%). Treatment with 1 mM methyl viologen was performed for 24 h by including this compound in the irrigation solution.

Experimental replicas of five pots per genotype and per treatment were used. Replicas were randomly distributed in the growth chamber to rule out possible variations due to small differences in environmental conditions within the growth chamber.

### Northern blot

Total RNA was isolated from drought stressed (Dehydration, DH), and methyl viologen (MV) treated plants, and from non-stressed control plants (Ctrl) of soybean N7001 and TJS2049 cultivars, using standard procedures. Ten µg of total RNA were separated in denaturing formaldehyde agarose gels and transferred onto nylon membranes (Hybond XL, Amersham Pharmacia Biotech). Ethidium bromide staining of the gels was used to ensure that equal amounts of RNA were loaded in the gels. Membranes were prehybridized at 65°C in 5X SSPE, 5X Denhardt’s solution, 0.2% SDS and 0.5 mg/mL denatured salmon sperm DNA, and hybridized overnight at 65°C with 50 ng of DNA probe, labeled with [α-32P] dCTP using Amersham Rediprime II DNA Labeling System (GE Healthcare Life Sciences), and purified by G-25 columns from GE Healthcare. Radioactive membranes were exposed overnight and revealed in a Phosphorimager.The probe consisted on an exon sequence spanning the positions nt. 111 to nt. 593 of *CV1*. This region shares 93% identity with the corresponding sequence of CV2.

### RT-qPCR

For cDNA synthesis, 2 µg of total RNA were reverse transcribed with QuantiNova Reverse Transcription (RT) Kit (Qiagen, Germany). To estimate amounts of cDNA templates of the selected genes, quantitative RT-PCR assays were performed using specific primers listed on **Table S1**, designed by Primer3Plus (Untergasser et al., 2012). q-PCR was performed in an Applied Biosystems StepOne real time PCR system. Each 10 µL reaction contained 5 µL of SYBR Green PCR Master mix (2 X), 0.5 µM primers mix and 2 µL of template cDNA (1/10 dilution). The thermocycler was programmed to run for 5 min at 95°C, followed by 40 cycles of 15 s at 94°C, 30 s at 60°C. Transcript accumulation of each gene was normalized relative to the constitutively expressed Elongation Factor 1 B (Glyma.02G276600) gene. Amplification efficiencies of the different primer combinations were all > 90%. Relative expression was determined using the 2^-ΔΔCt^ method (Livak and Schmittgen, 2001). Each data point is the mean value of three biological replicates. Two technical replicates were used for each sample.

### Phylogenetic analysis

The deduced amino acid sequences from *CV* genes were retrieved from the Phytozome 13 database. Sequences were aligned with ClustalW and phylogenetic analysis was done using MEGA 6 software (Tamura et al., 2013). Phylogenetic tree was done using the Neighbor joining method. The search on the gymnosperms, carnivorous plants and genomes outside plant kingdom was performed using protein BLAST and tBLASTn at NCBI database.

### Constructs for promoter activity analysis

Constructs for promoter activity analysis of *CV1* and *CV2* were generated by cloning the1500 bp promoter sequences upstream of the start codon of each gene into pBGWFS7 vector (Karimi et al., 2002), to regulate GUS expression. Promoter sequences were PCR amplified from genomic DNA of soybean TJS2049, using Kapa HiFi Hotstart DNA polymerase. Fragments of approximately 3000 bp were amplified using primers Fw 5’-CATAGATACTCATCGAAAATGG-3’ and Rv 5’-GTTGAGGATGTTTTGGGGGAA-3’ for Pro-*CV1*, and Fw 5’-GCCAAGTCCCAAAAGTGAACA-3’ and Rv 5’-GTGGCTTTGCGGGGTTGAAAGAAGCGT-3’ for Pro-*GmCV2*. *Bam*HI and *Xho*I restriction sites were added to 1500 bp fragment through nested PCR using primers Fw 5’-AATGGATCCTTATAGATGATAAAACAG-3’ and Rv 5’-TGGTCTCGAGGGTGAGAGTTGAGTTGAG-3’ for Pro-*CV1* or Fw 5’-AATGGATCCTTATTTTGGTCAGGGATT-3’ and Rv 5’-TGGTCTCGAGGGTGAGAGTTGAGACAAT-3’ for Pro-*GmCV2*. PCR fragments were cloned into *Bam*HI and *Xho*I restriction sites in pENTR-2B (Invitrogen-Thermo Scientific) and sequenced. Clones were subsequently recombined into pBGWFS7, using Gateway LR Clonase II (Invitrogen-Thermo Scientific). The constructs were introduced into *Agrobacterium tumefaciens*, strain C58C1 (Deblaere et al., 1985) by electroporation and used for Arabidopsis transformation.

### Constructs for overexpression

For the generation of constructs for conditional overexpression of CV1 and CV2, cDNA was synthesized from total RNA samples of soybean TJS2049 exposed to dehydration stress. Two µg of total RNA was treated win DNAseI and reverse transcribed into cDNA using oligo(dT) primer and RevertAid reverse transcriptase (Thermo Fisher Scientific). Coding sequences (CDS) were PCR amplified using Kapa HiFi Hotstart DNA polymerase and the primers Fw 5’-ATAAATTTTCTCAACTCAACT-3’ and Rv 5’-CATAAATCCAATCCAGTACTAG-3’ for *CV1* or Fw 5’-TCCAACCTATAACTCTAACCA and Rv 5’-AAAAAAAAACGTGACATATCA-3’ for *CV2*. *Bam*HI and *Xho*I restriction sites were added through nested PCR using primers Fw 5’-TCAACGGATCCC ATGAGGACCAGTTGCTTCC-3’ and Rv 5’-TTAGCTCGAGTCACATGGAGAAGCAACT-3’ for *CV1* or Fw 5’-ACTGGATCCATGAGGACCACTTGCTTA-3’ and Rv 5’-TGCGCTCGAGTCACATGGAGAAGCAGCTG-3’ for *CV2*. CDSs of each gene were cloned into *Bam*HI and *Xho*I restriction sites of pENTR-2B vector. The resulting clones were fully sequenced and further recombined into pHb7m34GW vector in combination with β-estradiol inducible promoter (p1R4-pG1090:XVE) and FLAG epitope (pEN-R2-3xFLAG-L3), using Gateway LR Clonase Plus (Invitrogen-Thermo Scientific). The constructs were introduced into *Agrobacterium tumefaciens*, strain C58C1 (Deblaere et al., 1985) by electroporation and used for Arabidopsis transformation.

### Arabidopsis transformation and molecular characterization of transgenic lines

Transgenic Arabidopsis plants were generated by *Agrobacterium* mediated floral-dip transformation (Clough and Bent, 1998). Transgenic lines were identified by PCR amplification using specific primers for *CV1* or *CV2* promoters or CDS-FLAG fusions. For promoter analysis, primers Fw 5’-AATGGATCCTTATAGATGATAAAACAG-3’ and Rv 5’-TGGTCTCGAGGGTGAGAGTTGAGTTGAG-3’ for Pro*CV1* or Fw 5’-AATGGATCCTTATTTTGGTCAGGGATT-3’ and Rv 5’-TGGTCTCGAGGGTGAGAGTTGAGACAAT-3’ for Pro*CV2* were employed. For CDS-FLAG fusions, primers Fw 5’-GGTGGCGCCAAATGGAGCG-3’ and Rv35STer 5’- AAGTGCGAGCGTAAAGGATAG-3’ for CV1-FLAG or Fw 5’-TGGTGGCGCCAAATGGAGCC-3’ and Rv35STer for CV2-FLAG were used. For CV1 or CV2-FLAG lines, expression of the transgenes was confirmed in three independent T2 lines by semiquantitative RT-PCR after treatment of plants with 5 µM β-estradiol (Sigma-Aldrich, E2758) using primers Fw 5’-GGTGGCGCCAAATGGAGCG-3’ and Rv35STer 5’- AAGTGCGAGCGTAAAGGATAG-3’ for CV1-FLAG or Fw 5’-TGGTGGCGCCAAATGGAGCC-3’ and Rv35STer for CV2-FLAG. Expression of Arabidopsis actin 2 gene (AT3G18780) was used as an internal control, by RT-PCR amplification with primers F: 5’-GCAACTGGGATGATATGGAAAAGA-3’, and R: 5’-TTTGTGGGAATGGAAGCTGCT-3’.

### *Arabidopsis thaliana* experimental conditions

For promoter expression analysis, Arabidopsis transgenic seedlings were grown *in vitro* for 5 to 7 days and exposed to abiotic stress for 3 days. Seedlings were transferred to Petri dishes containing MS supplemented with 150 mM NaCl (salt stress) or 2 µM methyl viologen (oxidative stress), 40% polyethylene glycol (PEG) 8000-infused agar plates (osmotic stress) and liquid MS with no air pumping (hypoxia). For hormone treatments, seedlings were transferred to MS supplemented with 50 µM ABA (abscisic acid), 100 µM ACC (aminocyclopropane-1-carboxylic acid), 10 µM gibberellin (GA3), 0 to 2 mM salicylic acid (SA) or 0 to 1 µM indole acetic acid (IAA). For methyl jasmonate (Me-JA) treatment, a cotton swab embedded in a 30 µM Me-JA solution was introduced into the plate which was tightly sealed with plastic wrap during treatment. An equal dilution of ethanol was used as control. Treatments with cytokinin were performed as follows: seven-day-old *in vitro* grown seedlings were transferred to peat moss and grown for 10 more days under 16/8 light/dark photoperiod. Plants were subsequently transferred to dark for 5 days and were both watered and sprayed with 5 µM benzylaminopurine (BAP) or only water. Control plants were kept in 16/8 light/dark photoperiod and were treated with 5 µM BAP or water. GUS activity was monitored by histochemical analysis as described by Vitha et al. (1995), or by fluorimetric assays as described by Cervera (2005). Experiments were repeated five times. Two or three independent lines for each promoter construct were analyzed in every experiment.

### Immunoblot assays

One hundred and twenty mg of tissue of Arabidopsis transgenic lines overexpressing CV-FLAG were frozen in liquid N_2_ and grounded using a bead beater homogenizer. Proteins were extracted in an extraction buffer (100 mM Tris-Cl pH 7.5, 100 mM NaCl, 1 mM EDTA, 2% SDS, 1% protease inhibitor cocktail Sigma Cat. no. P9599) in a 4:1 w/v ratio. Samples were centrifuged at 16000 x g for 10 min at 4°C. Supernatants were transferred to fresh eppendorf tubes and stored at -80°C until use. Thirty µg of total proteins were separated by SDS-PAGE, transferred to a Hybond-N+ nylon membrane (GERPN203B), and probed as described by Wang and Blumwald (2014). Antibodies raised against PsbO (AS05092), PsaB (AS06166A), PsbA/D1 (AS05084), GS1/GS2 and Lhcb2 (AS01003) were from Agrisera (Vännäs, Sweden). Antibodies raised against FLAG were from Sigma Aldrich (SAB4301138). IRdye 800 goat anti-rabbit secondary antibodies were obtained from LI-COR (926-32211). Horseradish peroxidase-conjugated goat anti-mouse secondary antibodies were purchased from Abcam (ab789).

### Statistical analysis

Statistically significant differences were determined based on the Student’s t-tests.

## Results

### CV genes are differentially regulated in drought sensitive or tolerant soybean genotypes

We have previously generated a subtracted cDNA library enriched in drought-induced genes from N7001 or TJS2049 genotypes (Gallino et al., 2018). Among the differentially regulated genes identified in this study, we found a CV encoding gene (Glyma.06G072000), designated as *CV1*.

**Figure 1:**
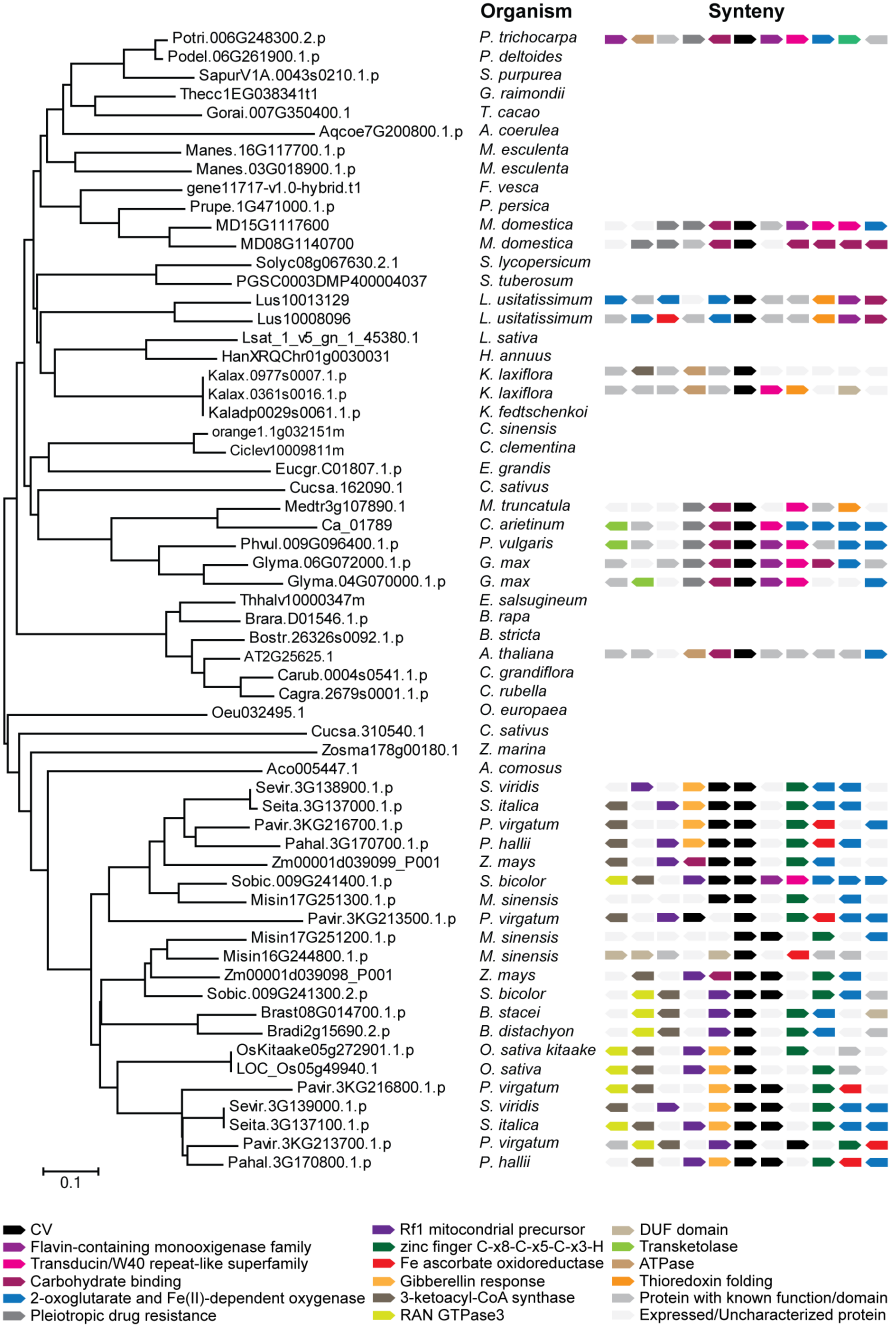
Phylogenetic and synteny analysis of CV proteins from angiosperms: *Glycine max, Populus trichocarpa, Populus deltoides, Sarracenia purpurea, Gossypium raimondii, Theobroma cacao, Aquilegia coerulea, Manhiot esculenta, Fragaria vesca, Prunus persica, Malus domestica, Solanum lycipersicum, Solanum tuberosum, Linum usitatissimum, Latuca sativa, Helianthus annuus, Kalanchoe laxiflora, Kalanchoe fedtschenkoi, Camellia sinensis, Citrus clementina, Eucaliptus grandis, Cucumis sativus, Medicago truncatula, Cicer arietinum, Phaseolus vulgaris, Eutrema salsugineum, Brassica rapa, Boechera stricta, Arabidopsis thaliana, Capsella grandiflora, Capsella rubella, Olea europaea, Zostera marina, Ananas comosus, Setaria viridis, Setaria italica, Panicum virgatum, Panicum hallii, Zea mays, Sorgum bicolor, Miscanthus sinensis, Brachypodium stacei, Brachypodium distachyon* and *Oryza sativa*. Full-length amino acid sequences were aligned by the ClustalW and a phylogenetic tree was constructed by the neighbor-joining method using MEGA version 6. Accession numbers of the genes are indicated in the figure. The two CV proteins of soybean (Glycine max) are located next to each other and display similar synteny between them and with respect to CV proteins in other legume species, which indicates that their genes were generated by duplication.

*CV* genes have not been found in any organism outside the angiosperm plant group, including chlorophytes, bryophytes, and the gymnosperms sequenced till date (**Table S2**). The soybean genome contains an additional CV encoding gene, *CV2* (Glyma.04G070000), which was not present in the cDNA library. According to phylogenetic and synteny analysis, *CV1* and *CV2* are paralogous that appeared through gene duplication (**Figure 1**). Interestingly most angiosperms sequenced so far contain a single copy of the *CV* gene, and in the case of legumes, soybean and trifolium are the only species containing two copies (**Table S2**).

In order to assess the contribution of the CV-mediated vesicular pathway to soybean responses to water deficit, we used northern blot to determine the expression profile of *CV1* in leaves and roots from drought sensitive (TJS2049) and slow wilting tolerant (N7001) genotypes exposed to dehydration or oxidative stress. Dehydration conditions were imposed by withholding irrigation until maximum substrate water retention capacity reached 25% while non-stressed control plants continued to be daily irrigated at 100% substrate capacity.

We also monitored *CV1* expression profile in response to oxidative stress, as this is a secondary stress condition in plants exposed to water deficit. Methyl viologen (MV) was used to induce oxidative stress by promoting the accumulation of superoxide anion radicals in chloroplasts, and *CV1* transcript accumulation was monitored 24 h after treatments.

**Figure 2:**
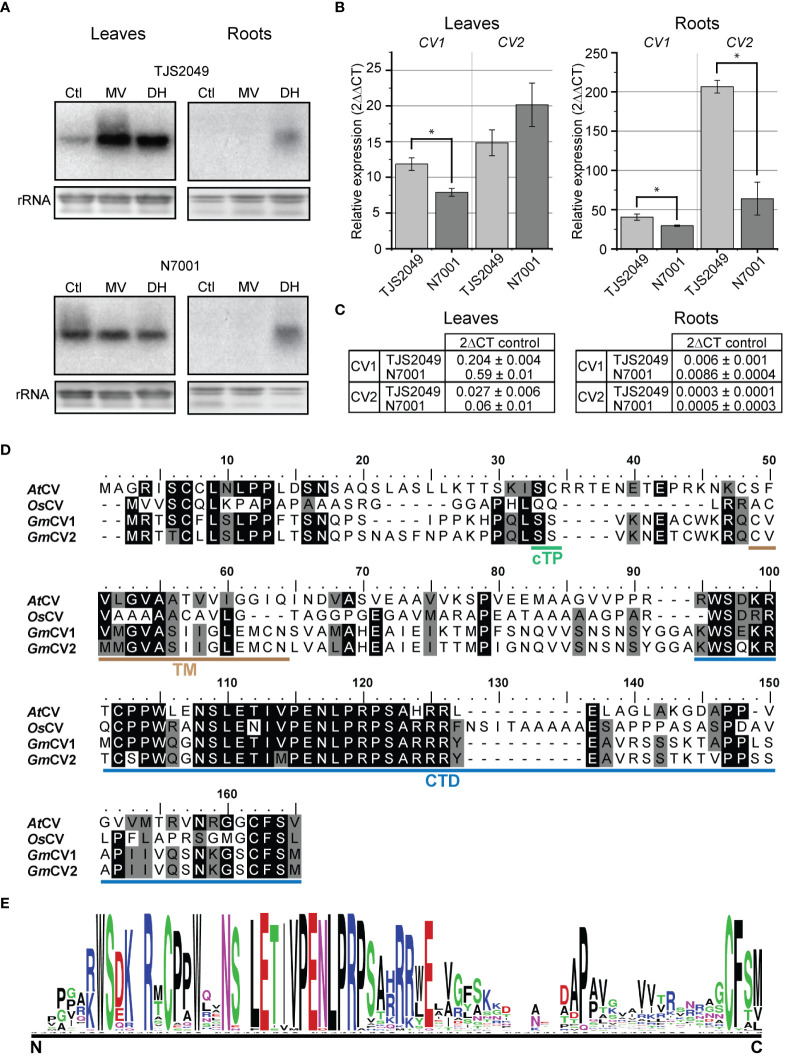
**(A)** *CV* transcript accumulation in leaves and roots of soybean TJS2049 and N7001 contrasting genotypes. Plants were treated with 1 mM methyl viologen (MV) for 24 h, or allowed to dehydrate (DH) until reaching 25% of water in the substrate. Total RNA was extracted from untreated control plants (Ctl) or treated plants. Ten μg of RNA were analyzed by northern blot using a ^32^P-labeled hybridization probe of *CV1*. Ethidium bromide staining of ribosomal RNA (rRNA) was used to ensure equal loading of RNA samples. **(B)** RT-qPCR analysis of *CV* genes under DH condition. Transcript accumulation of each gene was normalized relative to the constitutively expressed *Elongation Factor 1* **(B)** Each data point is the mean value of three biological replicates. Statistically different values are indicated with asterisks. Two technical replicates were used for each sample. **(C)** Relative expression of *CV* transcripts (2^-∆CT^) with respect to *Elongation Factor 1 B* in control condition in leaves and roots. **(D)** Alignment of CV proteins from soybean (GmCV1 and GmCV2), Arabidopsis (AtCV) and rice (OsCV), was performed using ClustalW. Cleavage site for chloroplast transit peptide was predicted using TargetP2.0 and is indicated in green (cTP). The transmembrane domain, predicted by LOCALIZER, is indicated in brown (TM) and the conserved C-terminal domain (CTD) is indicated in blue. **(E)** Conserved C-terminal domain logo weblogo (https://weblogo.berkeley.edu/logo.cgi).

The results showed that both dehydration and oxidative stress conditions induced higher levels of transcript accumulation of *CV1* in TJS2049 than in N7001 (**Figure 2A**), suggesting a larger contribution of the CVV pathway in the stress response of the sensitive cultivar compared to that of the slow wilting genotype. Interestingly, *CV1* was also significantly induced upon dehydration treatment in roots from both TJS2049 and N7001, indicating a role for this pathway in non-photosynthetic tissues.

Soybean *CV1* and *CV2* genes share 93% nucleotide sequence identity within the DNA fragment that was used as hybridization probe for northern blots. Therefore, it is likely that the hybridization signals observed in the northern analysis evidenced the combined accumulation of both *CV1* and *CV2* transcripts.

In order to assess the specific contribution of *CV1* and *CV2* in the response to dehydration of TJS2049 and N7001 contrasting genotypes, transcript accumulation of these genes was analyzed by quantitative real‐time polymerase chain reaction (qRT‐PCR), from leaves and roots tissue samples of plants exposed to dehydration conditions or grown under optimal conditions (**Figure 2B**). Induction levels were expressed as fold change by comparing drought-stressed versus well-watered controls. Consistent with the northern blot analysis, both genes were upregulated in TJS2049 and N7001 in response to water deficit, but the induction levels were higher in TJS2049 than in N7001. The higher stress-induced levels for both genes were observed in roots, where *CV1* transcript accumulation reached 40.6 ± 4.0 fold in TJS2049 and 29.7 ± 0.9 in N7001, and *CV2* transcript accumulated in 206.6 ± 8.1 in TJS2049 and 64.0 ± 20.9 in N7001. Stress-induction of *CV1* in leaves was considerably higher in TJS2049 than in N7001, whereas no significant differences were observed for *CV2*. Despite the fact that *CV2* showed higher fold change levels than *CV1*, the most actively expressing gene was found to be *CV1*, which had at least 10 times higher basal expression levels in non-stressed control plants (**Figure 2C**). Taken together, these results suggest that *CV1* has a larger contribution to stress-induced CVV pathway than *CV2* at the whole plant level, and that this route is predominantly induced in the sensitive genotype.

The biological function of CV has been characterized in detail in Arabidopsis (Wang and Blumwald, 2014), where it has been demonstrated that the protein accumulates in leaf chloroplasts under a number of abiotic stress conditions. The deduced amino acid sequence of *CV1* and *CV2* share 39/53% and 35/53% sequence identity/similarity with Arabidopsis CV, respectively. Like in Arabidopsis, soybean CV encoded proteins are predicted to contain a chloroplast transit peptide at the N-terminus, a transmembrane domain and a C-terminal conserved domain (**Figure 2D**). The graphical representation of the similarity patterns within CV encoded proteins from soybean, Arabidopsis and rice strongly suggests functional homology between these proteins (**Figure 2E**).

### Differential regulation of vesicular pathway marker genes involved in leaf senescence

In order to gain insight into the contribution of other vesicular routes involved in stress-induced senescence in soybean, we analyzed the expression profile of different marker genes for autophagy, SAVs and ATI-PS vesicular pathways in N7001 and TJS2049, under optimal conditions or exposed to dehydration stress. Because of the well-established role of CKs on counteracting natural or stress-induced leaf senescence (Sade et al., 2018), we also compared the expression profile of a CK response marker gene (*RR9*) in soybean contrasting genotypes in response to water deficit.

For the selection of marker genes, we took advantage of the previously generated cDNA subtracted libraries (Gallino et al., 2018) and from the reported RNA-seq (Shin et al., 2015) and microarray data (Le et al., 2012; Ha et al., 2015) of soybean genes differentially regulated under dehydration conditions (**Table S3**). In addition, we carried out in silico analysis of the expression profile of genes encoding representative members of autophagy, SAVs and ATI-PS pathways using available expression data at Genevestigator (Hruz et al., 2008), and found a number of genes that were upregulated in response to dehydration conditions (**Figure S1**). Based on these studies, the genes encoding *ATG8j* and *SAG12* (Glyma.17G239000), were selected as markers for monitoring the activation of autophagy and SAV vesicular pathways, respectively. In addition, the stress-induced *ATI1* gene (Glyma.09G258600), and the response regulator *RR9* gene (Glyma.11G155100), were selected as markers for ATI-PS pathway and for CK primary response, respectively. These genes were selected because they were also shown to be differentially regulated between TJS2049 and N7001 genotypes (Gallino et al., 2018).

Expression of the selected marker genes was used as a readout for the detection of different senescence-associated vesicular pathways in plants exhibiting contrasting phenotypes after exposure to dehydration conditions. RNA samples were extracted from N7001 and TJS2049 genotypes grown until the V2 stage and exposed to dehydration until reaching 25% of the substrate water retention capacity, or in plants that were kept irrigated (controls). Transcript accumulation levels were assessed by qRT-PCR and fold changes were calculated.

**Figure 3:**
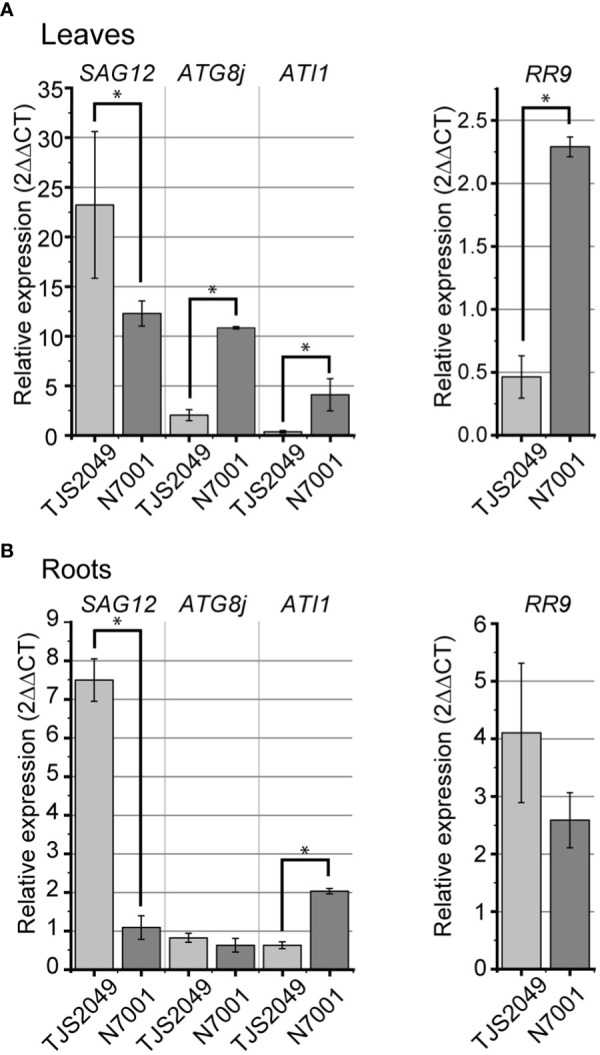
Relative mRNA expression levels of marker genes for autophagy, SAVs and ATI-PS pathways in soybean contrasting genotypes under dehydration conditions. RT-qPCR analysis of *SAG12* (Glyma.17G239000), *ATG8j* (Glyma.11G031800), *ATI1* (Glyma.09G258600) and *RR9* (Glyma.11G155100) under dehydration conditions (DH) in leaves **(A)** or roots **(B)** of soybean sensitive (TJS2049) or tolerant (N7001) plants. Transcript accumulation of each gene was normalized relative to the constitutively expressed *Elongation Factor 1* **(B)** Each data point is the mean value of three biological replicates. Statistically different values are indicated with asterisks. Two technical replicates were used for each sample.

All of the vesicular pathway marker genes were upregulated in response to water deficit regardless of the genotype, both in leaves and roots. However, the genes exhibited differential induction levels between slow-wilting and drought-sensitive soybeans. Similar to *CV* genes, *SAG12* exhibited higher induction levels upon dehydration in TJS2049 than in N7001 plants, while *ATG8j* and *ATI1* showed the opposite expression pattern. *ATG8j* only showed a strong induction in leaf tissues, with a fold change of 2.0 ± 0.6 for TJS2040 and 10.9 ± 0.1 for N7001. Induction of *SAG12* was stronger in TJS2049 than in N7001, reaching a fold change of 23.2 ± 7.4 for TJS2049 and 12.3 ± 1.3 for N7001. Similar responses were evidenced in roots for *SAG12*, reaching a fold change of almost 8 in TJS2049 while only a 2-fold induction in N7001. *ATI1* reached the maximum level of induction in the leaves of N7001 showing a 4.5fold increase, while no significant induction of this gene was observed in TJS2049 leaves (**Figure 3**).

Overall, marker genes for vesicular pathways with remobilization functions (autophagy and ATI-mediated) reached higher induction levels under stress conditions in the drought tolerant N7001 genotype than in TJS2049, which is in agreement with a pro-survival role of these pathways. In contrast, *SAG12* and *CV* genes reached higher induction levels in the drought-sensitive cultivar (**Figures 2B**, **3**). Noteworthy, the induction levels of *ATG8j*, *SAG12* and *ATI1* were overall lower than those exhibited by *CV1* gene in both plant genotypes (**Figure 2B**), suggesting a major role for *CV1* in stress-induced senescence in soybean.

Taken together, these results seem to indicate that stress-induced senescence was more installed in the TJS2049 genotype than in N7001. Consistent with these results, the CK response gene *RR9* was downregulated in leaves of TJS2049 exposed to water deficit, whereas this gene was upregulated in leaves from the N7001 genotype. This result is consistent with the delayed stress-induced leaf senescence phenotype exhibited by N7001.

### Differential expression patterns of *CV1* and *CV2* gene paralogous under abiotic stress and natural senescence

In order to determine whether soybean *CV* gene paralogous have undergone functional specialization, or on the contrary, they have maintained redundant functions, *in silico* analysis of their promoter regions was carried out to identify potential polymorphisms affecting promoter activity.

**Figure 4:**
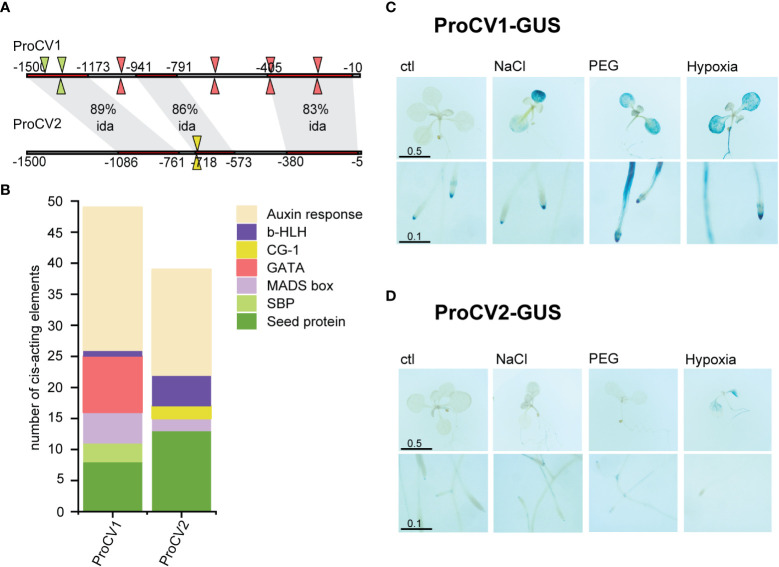
Promoter analysis and activity under abiotic stress. **(A)** CV1 and CV2 1500 bp promoter regions were aligned using Align Sequences Nucleotide BLAST tool at NCBI website. Shaded regions correspond to sequence with high sequence identity (ida). Cis-acting elements in the promoter regions were analyzed using PlantPAN3.0 (Chow et al., 2019). Triangles indicate cis elements that are only present in one of the promoter regions. Triangle colors correspond to the type of cis element according to **(B)**. **(B)** Abundance of different cis elements in promoters of CV1 and CV2. **(C, D)** GUS staining of 10-day-old transgenic plants containing ProCV1-GUS **(C)** or ProCV2-GUS **(D)** constructs subjected to 3 days stress treatment. In vitro grown transgenic seedlings were transferred to Petri dishes containing MS supplemented with 150 mM NaCl, 40 % PEG 8000-infused agar plates or liquid MS with no air pumping. The length of the scale bars is in centimeters.

Despite the high sequence identity shared by *CV1* and *CV2* at their coding regions, less conservation was observed at their promoter regions, considering the first 1500 bp upstream of the translation initiation codon. It was assumed that this region included the promoter elements that are necessary for *CV* gene regulation. The promoter sequences of *CV1* and *CV2* (proCV1 or proCV2, respectively), have 3 highly conserved regions and 2 regions with low conservation (**Figure 4A**). Regardless of the position within the promoter sequence, important differences were observed in the presence and abundance of cis-acting elements of known transcription factors involved in stress responses (**Figure 4B**). GATA transcription factor and SQUAMOSA PROMOTER BINDING PROTEIN (SBP) elements appeared in proCV1 but were absent in proCV2. On the other hand, a number of CG-1 elements, absent in proCV1, were present in proCV2. Important differences were also observed in auxin response elements, being more abundant in proCV1 than in proCV2. In contrast, b-HLH elements were more abundant in proCV2 than in proCV1. In addition, proCV2 displayed a higher number of elements characteristic of seed storage proteins (RY repeat, SEF1 and SEF4).

To gain experimental evidence on the differential promoter activities of *CV1* and *CV2*, the 1.5 kb promoter regions of these genes, were PCR-amplified from TJS2049 genomic DNA, cloned upstream of a promoterless GUS reporter gene and used for Arabidopsis transformation. The ability of soybean proCV1 and proCV2 to control GUS expression under optimal growth conditions or in response to different stresses was examined in stable Arabidopsis transgenic plants. We used Arabidopsis because of the advantages of this plant species (small size, short life cycle) compared to soybean, for monitoring tissue-specific *CV* expression patterns simultaneously in all tissues, as well as during the entire plant life cycle.

We analyzed specific GUS activity of *in vitro* grown Arabidopsis transgenic seedlings exposed to salt stress (150 mM NaCl), osmotic stress (40% PEG) or hypoxia (submersion in liquid MS without air pumping). Histochemical staining of whole plants was performed after 3 days of abiotic stress-treatments or in untreated control plants. At least 3 independent transgenic lines transformed with the same construct were analyzed for GUS activity in parallel experiments (**Figures 4C, D**).

Consistent with the RT-qPCR analysis of *CV1* and *CV2* expression profiles in soybean, these two genes exhibited significant differences in promoter activity under stress conditions when analyzed in the Arabidopsis heterologous system. ProCV1-drived GUS activity was triggered in shoots and roots of plants exposed to all abiotic stress conditions (**Figure 4C**). ProCV1 induced GUS expression in whole leaves and roots, particularly at the root tip, including the columella cells and the lateral root cap, as well as at the maturation zone of the principal root and at lateral roots. On the contrary, GUS expression resulting from ProCV2 activity was only evident after hypoxia stress, a condition that typically triggers generalized senescence in plants. (**Figure 4D**). Under these conditions, ProCV2-GUS activity was observed in roots and in the vascular areas of shoots, but in all cases, GUS staining signal was markedly weaker in these plants than in ProCV1-GUS transgenic plants. Together, these results support the idea that stress-induced accumulation of CV relies primarily on *CV1* gene expression.

In view of the current knowledge of the role of *CV* genes from Arabidopsis and rice in natural senescence, we explored the promoter activities of soybean *CV1* and *CV2* during different developmental stages and cues.

**Figure 5:**
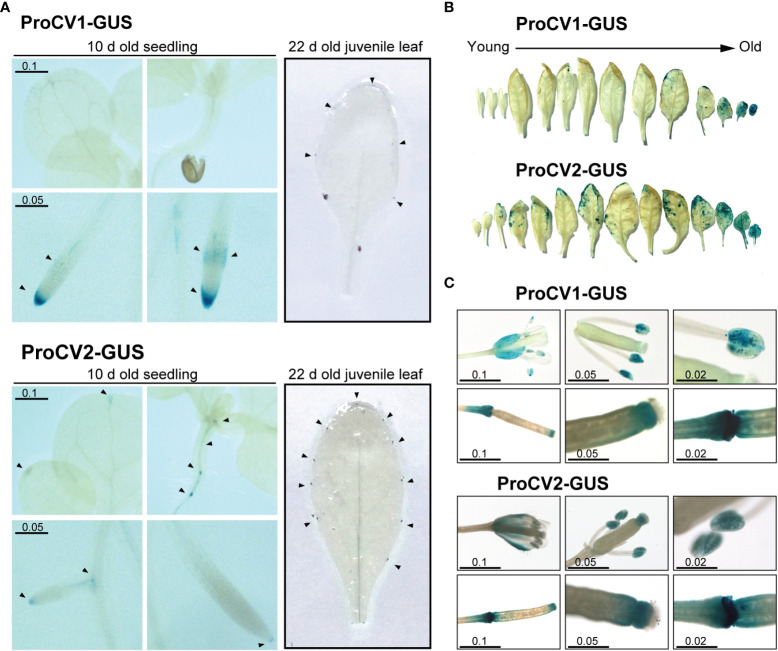
GUS activity in transgenic Arabidopsis plants during different developmental stages. **(A)** Ten-day-old seedling and 22day-old juvenile leaves or plants grown under optimal conditions, containing ProCV1-GUS or ProCV2-GUS constructs. Black triangles indicate regions showing strong GUS activity. **(B)** GUS staining of cassette leaves from representative 40-day-old transgenic plants. **(C)** GUS activity in flowers and siliques of transgenic plants. The length of the scale bars is in centimeters.

No significant differences on promoter activities of these genes were observed in early developmental stages of Arabidopsis transgenic plants grown under optimal conditions (**Figure 5A**). In shoots of young seedlings (10-days old), GUS activity driven by proCV1 or proCV2 was restricted to the leaf tip, in the mid vein, where differentiation of the vascular tissue (xylem) occurs (**Figure 5A**). In addition, GUS activity was also detected at the root vascular tissue, especially at the root’s central zone and in the root tip and lateral root primordia. On the other hand, in leaves of young juvenile transgenic plants (22-days old), promoter activity of both genes was observed in the tips of teeth during leaf serration formation. These structures define leaf shape and are regulated by auxin and several developmental cues (Tang et al., 2021).

We next investigated the specific contribution of *CV1* and *CV2* paralogous genes during natural senescence by assessing GUS activity throughout developmental progression (**Figure 5B**). A strong GUS signal resulting from *CV1* or *CV2* promoter activation was detected in mature senescent leaves, starting at the leaf margins, which coincided with the progression of natural senescence. Nevertheless, clear differences in the timing and the level of promoter activities of *CV1* and *CV2*, were observed. CV2 promoter was activated at earlier developmental stages, and to higher levels than proCV1, supporting a functional specialization of these genes during leaf senescence. In contrast, both *CV1* and *CV2* promoters exhibited similar activities in flowers and siliques, especially in sepals, anthers and the stigma, after petals and sepals abscission, and at the silique abscission zone (**Figure 5C**). Taken together, these results suggest that both genes play a role in tissues undergoing developmental processes involving cell death (root tip lateral root cap, vascular tissue development, abscission zones and natural senescence), but *CV2* has a larger contribution to the natural leaf senescence process than *CV1*.

### CK inhibits *CV2* expression under dark-induced senescence

Our results are consistent with the idea that soybean *CV1* and *CV2* gene paralogous have undergone a functional specialization process manifested through divergence in gene expression. While *CV1* is upregulated by abiotic stress, *CV2* appears to have a more predominant role in developmental-induced senescence. In Arabidopsis, CK delays senescence and suppresses *CV* gene expression (Matallana-Ramirez et al., 2013). Together, these data motivated us to investigate CK-regulation of *CV1* and *CV2* gene expression, by monitoring the promoter activity of these genes under dark-induced senescence of Arabidopsis transgenic lines, and assessing the effect of CK treatment on *CV* inhibition of gene expression.

**Figure 6:**
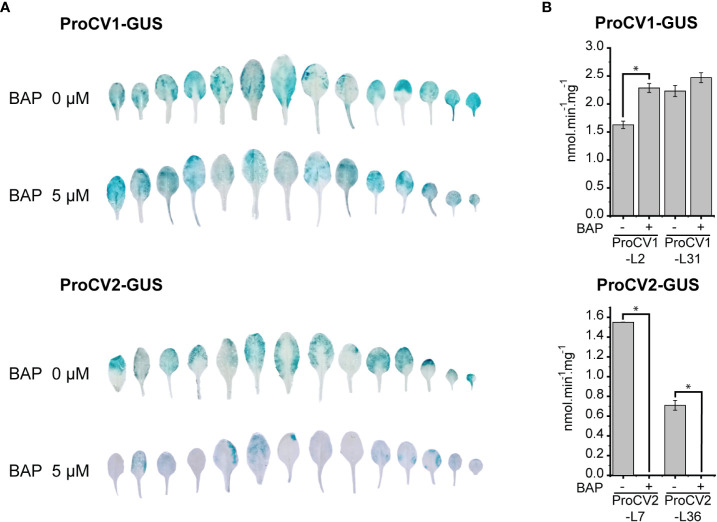
Effect of cytokinin treatments on CV1 and CV2 promoter activities in Arabidopsis transgenic plants exposed to darkness. **(A)** Transgenic plants (22 days old) containing ProCV1-GUS or ProCV2-GUS constructs were subjected to 5-day-light deprivation treatment, in the presence or absence of cytokinin (5 μM BAP). Histochemical assays were perform to detect GUS activity. **(B)** GUS specific activity measured by fluorimetric assays in leaves from same samples of two independent transgenic lines (L2 and L31), grown as A. Each data point is the mean value of three biological replicates. Statistically different values are indicated with asterisks.

Arabidopsis transgenic ProCV1-GUS and ProCV2-GUS lines were grown during 17 days under regular light conditions and thereafter transferred to darkness for 5 days, with or without exogenous CK (5 µM BAP). GUS staining assays showed that, in the absence of BAP, both *CV1* and *CV2* promoters were strongly activated during dark-induced senescence. However, clear differences in the regulation of *CV* genes were observed in the presence of exogenous BAP, where the activity of the *CV2* promoter was almost completely inhibited, while no inhibition was observed for *CV1* (**Figure 6A**). CK differential inhibition of *CV* genes was confirmed by quantitative analysis of GUS activity by performing fluorimetric assays in two independent representative transgenic lines for each construct (**Figure 6B**). Similar to Arabidopsis *CV* gene, soybean *CV2* appears to be strongly downregulated by CK under senescence-inducing conditions, whereas *CV1* promoter activity was unaffected by this hormone.

### Hormonal control of *CV* promoter activity

Plant hormones regulate central features of development and stress adaptation. In fact, most known plant growth regulators, such as auxin, ABA, CK, among others, have been shown to mediate different developmental and stress responses through complex crosstalk signaling pathways (Waadt et al., 2022).

**Figure 7:**
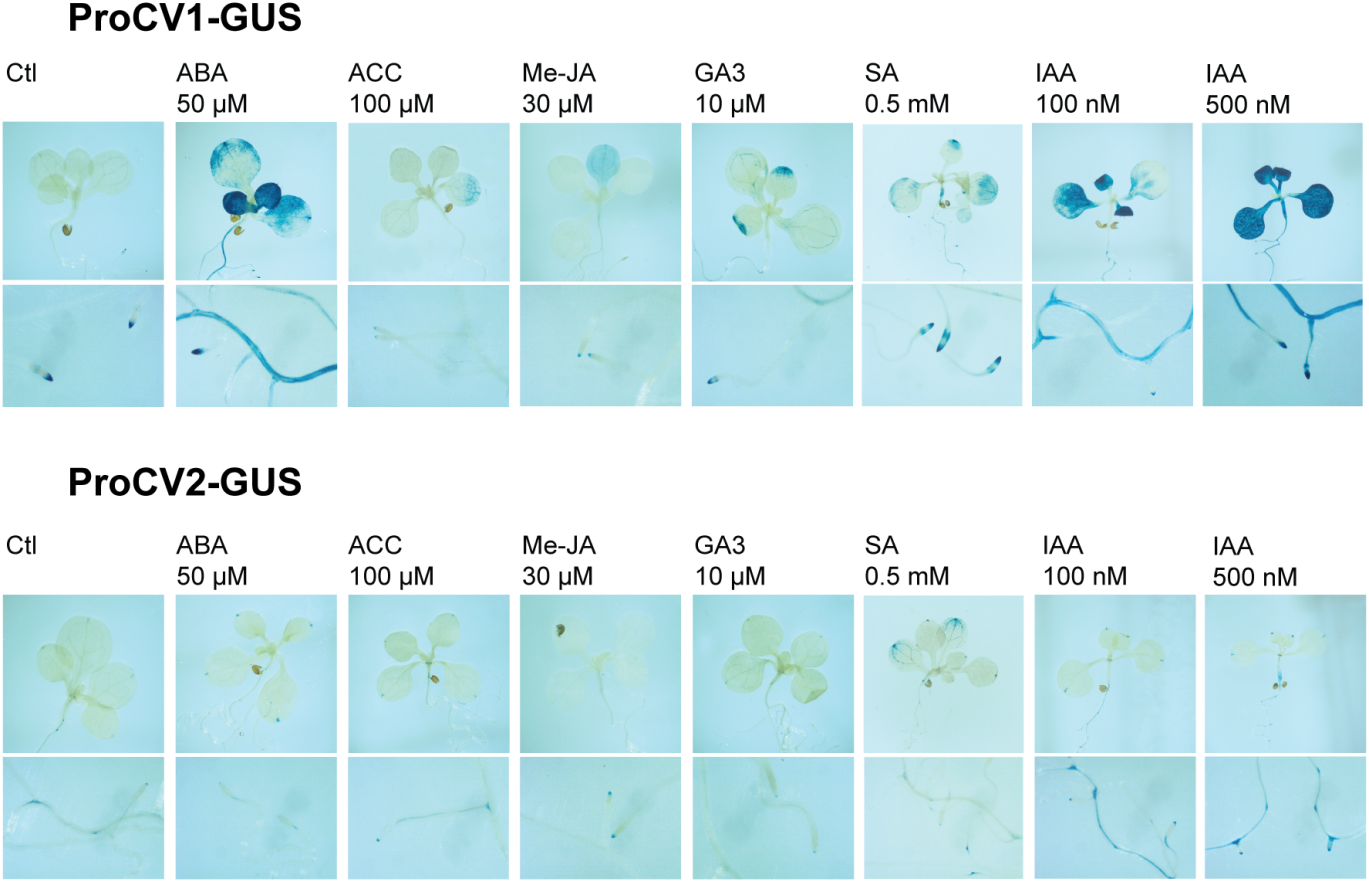
GUS activity in response to hormonal treatments. Seven-day old in vitro grown transgenic seedlings (ProCV1-GUS or ProCV2-GUS), were transferred to Petri dishes containing MS supplemented with 50 µM ABA, 100 μM ACC, 10 µM gibberellin (GA3), 0 to 2 mM salicylic acid (SA), and 100 nM or 500 nM IAA. For methyl jasmonate (Me-JA) treatment, a cotton swab embedded in a 30 μM Me-JA solution was introduced into the plate. Treatments were performed for 4 h and plants were subsequently processed for histochemical GUS assays.

To further investigate the regulation of *CV1* and *CV2* gene expression, we studied their specific promoter activity in response to the treatment of plants with different stress-related hormones. Arabidopsis transgenic plants expressing GUS reporter gene under the control of *CV1* or *CV2* promoters, were treated with 50 μM ABA, 100 μM ethylene precursor 1-aminocyclopropane-1-carboxylic acid (ACC), 30 μM methyl jasmonate (Me-JA), 10 μM gibberellic acid (GA3), 0.5 mM salicylic acid (SA) and 0.1 μM or 0.5 μM 3-indoleacetic acid (IAA). Histochemical GUS activity was monitored 4 h after hormone treatments (**Figure 7**).

All treatments induced GUS activity driven by proCV1, but the highest induction levels were observed in response to exogenous ABA or auxin (IAA), both in shoot and roots (**Figure 7A**). In contrast, significant proCV2 activity was only detected in roots of plants treated with auxin, and to some extent, in shoots after SA treatment (**Figure 7B**). Auxin regulation of both *CV1* and *CV2* was consistent with the presence of several auxin response elements in their promoter regions (**Figure 4B**), which were more abundant in *CV1* promoter sequence. The fact that only proCV1 responded to typical stress hormones, such as ABA, SA, Me-Ja and ACC, supports the role of this paralogous in stress responses.

### CV overexpression led to chloroplast protein degradation

**Figure 8:**
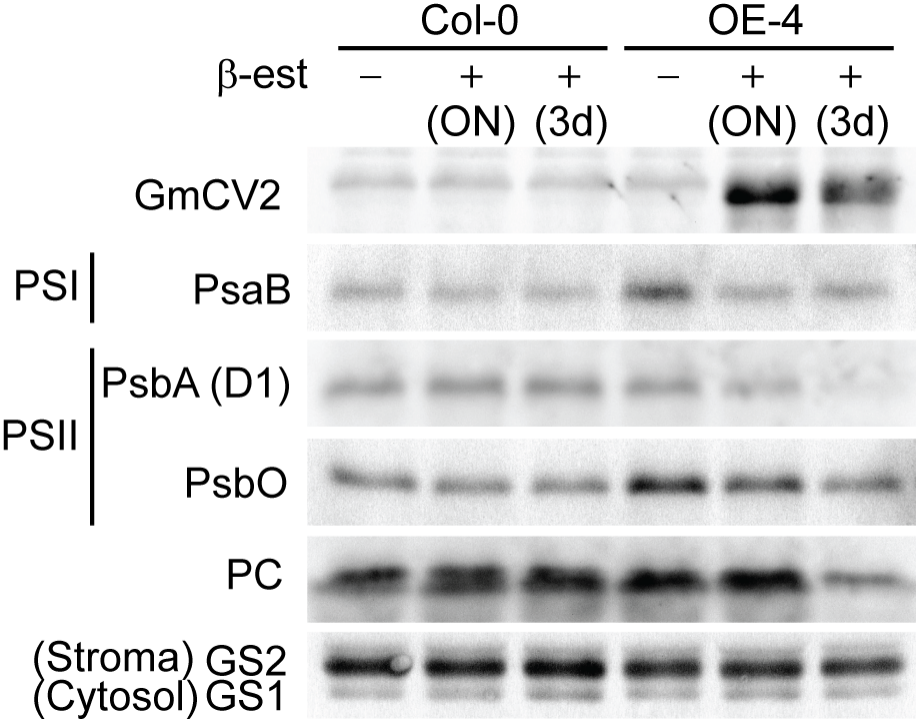
Conditional overexpression of CV2-FLAG results in chloroplast protein degradation. Protein samples from Arabidopsis wild type (Col-0) and transgenic plants overexpressing CV2-FLAG fusion protein under the control of a β-estradiol inducible promoter, were analyzed by western blot for the abundance of chloroplast-targeted proteins in the presence or absence of β-estradiol (β-est). Seven-day-old transgenic plants containing XVE-CV2-FLAG construct (OE-4) or Col-0 plants were transferred to Petri dishes containing MS supplemented with 5 µM β-estradiol or DMSO as control. Chloroplast proteins were detected with specific antibodies against PSI subunit PsaB, PSII subunits PsbA (D1) and PsbO1, stromal proteins plastocyanin (PC) and GS2. Cytosol protein is GS1. Antibodies against FLAG were used to detect CV2-FLAG fusion protein.

Except for the highly conserved C-terminus domain of CV proteins, the overall sequence similarity between CVs from different plant species is not higher than 50%. Despite this, functional conservation between CV proteins has been demonstrated, at least for Arabidopsis and rice genes. Given the features in soybean *CV* encoded proteins, including the presence of a chloroplast transit peptide, a transmembrane domain and a highly conserved C-terminus domain, it is presumably that soybean CVs have similar biological function as Arabidopsis and rice orthologs. To experimentally verify this assumption, we used a β−estradiol inducible overexpression system to drive the expression of CV2-FLAG fusions in transgenic Arabidopsis and analyzed the effect of CV2-overexpression in specific chloroplast protein levels. Two independent stable transgenic lines, exhibiting high CV2 transcript and protein accumulation after β-estradiol treatment, were selected for further characterization. Wild type and transgenic Arabidopsis plants were grown *in vitro* for 7 days and subsequently treated with 5 μM β-estradiol for 12 h (overnight) or for 3 days. Leaf protein samples from untreated controls or β-estradiol treated plants, were analyzed in immunoblot assays to determine the levels of PSI complex subunit PsaB, and PSII subunits D1 and PsbO, as well as the stromal glutamine synthase 2 (GS2) and plastocyanin (PC) (**Figure 8**). The results showed that overexpression of CV2 resulted in reduced levels of chloroplast proteins, especially PsaB, D1 and PsbO, confirming a functional conservation of soybean CV2 in promoting chloroplast protein degradation.

## Discussion

We previously established that the slow wilting N7001 genotype has a number of physiological traits associated with drought resistance, such as improved WUE and minimal losses in biomass accumulation, after exposure to drought conditions (Gallino et al., 2018). Comparative studies of the drought responsive transcriptomes of TJS2047 and N7001 genotypes showed significant differences in the expression of genes involved in different vesicular pathways associated with natural and stress-induced senescence (Gallino et al., 2018).

Here, we provide evidence for the differential contribution of specific senescence-associated vesicular pathways in plants exhibiting contrasting phenotypes in response to water deficit. Expression of marker genes was used as a readout for the detection of CVV (*CV1* and *CV2*), autophagy (*ATG8j*), SAV (*SAG12*) and ATI-PS (*ATI1*) pathways in N7001 and TJS49.

All the marker genes were upregulated in response to water deficit in leaves and roots of both N7001 and TJS2049 genotypes, but the induction levels differed significatively between genes and genotypes (**Figures 2B**, **3A, B**). Regardless of the genotype, *CV* genes exhibited the strongest expression levels, suggesting that the CVV pathway plays a prominent role in soybean responses to drought stress (**Figures 2B**, **3**).

Genotypic differences of *CV* and *SAG12* stress-induction levels were observed in root tissues, where these genes reached higher induction levels in TJS2049 than in N7001, suggesting a negative correlation between these pathways and drought tolerance in soybean. In contrast, *ATG8j* and *ATI1* were more induced in N7001 than in TJS2049, and these differences were observed both in leaves and roots (**Figures 2A, B**, **3**). Marker-gene expression profiles were consistent with the enhanced stress-induced senescence symptoms observed in TJS2049 genotype: a higher induction of CVV and SAVs pathways and a lower induction of autophagy and ATI-mediated degradation. While CVV and SAVs pathway are associated with recycling mechanisms typical of senescent tissues (Otegui et al., 2005; Wang and Blumwald, 2014; Sade et al., 2018), autophagy and ATI-mediated pathways have been shown to have also a pro-survival role by participating in recycling of damaged cellular components, avoiding the deleterious effects of damaged chloroplasts on cell viability (Chen et al., 2019). Thus, mechanisms involved in the control of the onset of stress-induced senescence may contribute to the slow wilting phenotype exhibited by N7001. Consistently, the higher stress-induction levels of *RR9*, a CK responsive gene, in leaves of N7001 (**Figure 3A**) supports a lower level of stress-induced senescence in this genotype.

As CV proteins are the central component of the CVV pathway for chloroplast degradation, we hypothesize that the low induction levels of these genes observed in the slow wilting cultivar, contribute to the drought resistance phenotype. Nevertheless, whether low levels of CV accumulation is causing drought resistance or it is simply a consequence of it, needs to be further clarified.

The strong upregulation of *CV* genes in root tissues indicated the possible role of this pathway in non-photosynthetic tissues. During leaf senescence, chloroplast degradation is an early event, which results in remobilization of nutrients and energy to developing tissues and storage organs, such as seeds (Avila-Ospina et al., 2014). In crop plants, N remobilization during seed maturation is highly correlated to grain yield and quality (Kichey et al., 2007). In legumes, roots are an important N source, but the role of vesicular pathways in N remobilization from roots is poorly understood. As a legume, soybean can establish a symbiotic association with N-fixing bacteria of the Rhizobium genus, which are housed in the root nodules. Drought inhibits nodulation and N fixation, and soybean yields are especially affected when drought occurs during flowering and during pod establishment at which time the nodules start showing senescence symptoms (Márquez-García et al., 2015). In drought tolerant soybean varieties, there is a strong correlation between root and nodule parameters and the biomass accumulated in the aerial part (Fenta et al., 2012; Fenta et al., 2014). Although N remobilization from the root to the seeds is poorly documented, a study in *Brassica napus* showed that more than 11% of the N in the seeds comes from the root (Rossato et al., 2001). These observations indicate that the root is an important source organ for grain filling during plant senescence.

The role of CV pathway in root senescence has not been previously explored, and little is known about the role(s) of other vesicular-mediated pathways in roots. In Arabidopsis, SAG12 protease has been shown to participate in root N remobilization to the seeds (James et al., 2019). In rice, autophagy has been shown to mediate root plastid degradation during energy limitation conditions (Izumi and Ishida, 2011). Also, this pathway is involved in amyloplast degradation of Arabidopsis during water stress (Nakayama et al., 2012), and in root architecture in response to osmotic stress and nutrient starvation (Slavikova et al., 2008). In addition, several ATG8 isoforms (ATG8c, ATG8d, ATG8g, ATG8h) have been related to xylem and phloem differentiation in roots and stems of *Populus trichocarpa* (Wojciechowska et al., 2019). Degradation of organelles also occurred in phloem cells, where the few that remain are located in a peripheral strand of cytoplasm along the cell wall (Knoblauch and van Bel, 1998). However, the mechanisms involved in this degradation process are largely unknown. Interestingly, *in silico* analysis of gene expression profiles of soybean gene families encoding ATG8, SAG12, ATI1 and CV, showed that, except for one member of the *SAG12* gene family, out of at least 43 genes, *CVs* were the only genes strongly upregulated by abiotic stress (salinity) in roots. (**Figure S1**). RT-qPCR analysis of genes encoding proteins associated with vesicular-mediated pathways confirmed that marker genes, other than *CVs*, showed very low induction levels in roots upon drought stress (**Figure 3B**). The biological significance of the CVV-mediated pathway in roots is not clear, but it is possible to speculate that CVs could be involved in stress-induced plastid degradation and further N remobilization.

Soybean genome has two *CV* paralogous genes that, based on synteny analysis, arose from a duplication event throughout evolution of this plant species (**Figure 1**). Gene expression divergences, resulting from changes in the regulatory regions, have been proved for a wide number of gene paralogs (Lian et al., 2020). The differential expression levels of *CV1* and *CV2* observed by RT-qPCR analysis suggested that CV1 played a more relevant role than CV2 in soybean responses to water deficit (**Figures 2B, C**).

Analysis of *CV1* and *CV2* promoter regions provided strong evidence that these genes have diverged from each other to participate in different biological processes. Our results showed that while *CV1* is primarily regulated by abiotic stress stimuli and stress-related hormones, *CV2* is for the most involved in developmentally programmed senescence and dark-induced senescence.

CV1 and CV2 deduced proteins share 88% sequence similarity (**Figure 2D**), suggesting functional redundancy of these proteins. However, their promoter regions differ in the presence and number of several regulatory elements (**Figure 4B**). For example, GATA and SBP response elements were present in *CV1* and absent in *CV2* promoter regions, whereas CG-1 elements were present in *CV2* and absent in *CV1* promoters. The *CV2* promoter also had a higher abundance of b-HLH and seed elements (RY repeat, SEF1 and SEF4). Transcription factors of the GATA, SBP, CG-1, b-HLH and MADS-box families are involved in the regulation of a wide variety of developmental processes as well as in responses to environmental stimuli (Finkler et al., 2007; Shen et al., 2015; Abdullah-Zawawi et al., 2021; Hao et al., 2021; Hussain et al., 2021; Schwechheimer et al., 2022). Furthermore, the presence of storage protein regulatory elements in *CV* promoters is consistent with a role of these genes during protein degradation processes occurring simultaneously to grain filling. Similar to *CVs*, a number of SAG genes have been shown to be expressed during seed maturation and germination, providing evidence for common mechanisms involved in these processes and leaf senescence (Smart et al., 1995). Experimental analysis of the promoter activities of *CV1* and *CV2* in Arabidopsis heterologous system confirmed the predicted differences in gene regulation. First, proCV1 was highly responsive to all stress conditions (salinity, hyperosmotic and hypoxia), both in leaves and in roots (**Figure 4C**), whereas proCV2 was only moderately activated in leaves under hypoxia (**Figure 4D**). In contrast, proCV2 exhibited earlier and higher activity than proCV1 under natural senescence (**Figure 5B**). Nevertheless, both *CV* promoters were responsive to dark-induced senescence, but only proCV2 activity was inhibited by CK under these conditions (**Figure 6**). Dark-induced senescence has many features common to developmental senescence. However, studies in both monocots and dicots have shown that there are considerable differences in gene expression between these processes (Balazadeh et al., 2008; Sobieszczuk-Nowicka et al., 2018). The observed differences in *CV1* and *CV2* gene expression during development, and their differential responsiveness to CK during dark-induced senescence, is consistent with the existence of specific regulatory mechanisms controlling these processes.

Marked differences in promoter activities were also observed after hormone treatments, where *CV1* promoter showed higher levels of hormone-induced activity in all cases (**Figure 7**). Remarkably, ABA treatment triggered *CV1* promoter activity, both in leaves and in roots, whereas *CV2* promoter was unresponsive. ABA regulation of *CV1* is consistent with Arabidopsis and rice expression profiles of *CV* genes (Peleg et al., 2011; Wang and Blumwald, 2014) and with a role for *CV1* in abiotic stress-induced senescence.

An interesting result was the auxin regulation of *CV* genes. Although both genes were upregulated in response to IAA treatment, the induction levels were significantly higher for *CV1* than *CV2* promoter regions. Moreover, IAA-induction of *CV1* promoter was observed in both leaves and roots, while only low levels of *CV2* promoter activity were detected in roots (**Figures 7**, **S2**). These results are consistent with the higher abundance of auxin response elements in *CV1* promoter compared to *CV2* promoter (**Figure 4B**).

Auxin is produced in young growing cells present in shoot tips, young leaves, and in developing flowers, seeds and roots (Ljun et al., 2002; Brumos et al., 2018). In addition, auxin accumulates in places where developmental programmed cell death (dPCD) is taking place, including anther dehiscence, root cap shedding, xylem differentiation and organ senescence (Sheldrake, 2021). The observed promoter activity of *CV1* and *CV2* in the root tip, lateral root primordia and leaf serrations of young non-stressed plants coincides with tissues with high endogenous auxin accumulation. In addition, high *CV1* and *CV2* promoter activity was observed in the abscission zones of the transgenic Arabidopsis floral organs, stamens, petals, and sepals of flowers, corresponding to tissues undergoing senescence or dPCD (**Figure 5C**).

The activity of *CV* promoters during natural senescence of the leaves, the flowers and the siliques is remarkably similar to the promoter activities of *BFN1*, *ORE1* and other NAC transcription factors in these organs (Matallana-Ramirez et al., 2013; Kim et al., 2014). Considering that binding sites for NAC transcription factors are present in both *CV* promoters and that there are no differences in the content of this particular cis-element, it could be speculated that NAC transcription factors might be upstream of *CV* expression during natural senescence.

The accumulation of GUS staining in the root tip and lateral root primordia for both *CV* promoters under non stress conditions (**Figure 5A**) suggests that CV proteins may be relevant for root development or architecture. It is interesting to note that this expression profile at the root tip overlaps to that of NAC transcription factors involved in dPCD control at the columella and lateral root cap, ANAC033 (SOMBRERO: SMB), ANAC087, and ANAC046 (Bennett et al., 2010; Fendrych et al., 2014; Huysmans et al., 2018). This type of PCD has been implicated in maintaining root cap organ size and position at the root tip which is important for meristem protection and root gravitropism (Kumpf and Nowack, 2015). Transcriptional activation at lateral root primordia is also coincident with activation of *ORE1* NAC transcription factor (ANAC092) involved in endodermis cell death and cell death marker genes (*BFN1, MC9, DMP4*, and *RNS3*, Farage-Barhom et al., 2008; Olvera-Carrillo et al., 2015; Escamez et al., 2020). Cell death at endodermis, cortex and epidermis is necessary for the elimination of cells overlying lateral root primordia (Wachsman and Benfey, 2020).

The auxin regulation of *CV* genes suggests that these genes are actively transcribed in auxin rich dying tissues, where they could participate in N remobilization as a consequence of plastid degradation. This supports the hypothesis of the auxin production in dying cells (Sheldrake, 2021) that proposes that auxin accumulates in dying tissues as a breakdown product of tryptophan, which is released as a consequence of protein degradation during cell death.

CV pathway is a route that participates in the degradation of thylakoid and stromal proteins independently of autophagy or SAVs. CV protein contains a peptide for chloroplastic localization and only appears to be linked to the chloroplast and to the CVVs. *CV* silencing in Arabidopsis resulted in chloroplast stability during stress and enhanced stress tolerance (Wang and Blumwald, 2014). Sade et al. (2018) confirmed these observations in rice plants in which the *OsCV* gene was silenced. RNAiOsCV plants showed intact grana and were able to maintain primary N assimilation processes and displayed better yield performance under water-deficit conditions. Results revealed the role of CV in the turnover of enzymes that are associated with N assimilation during stress, leading to decreased N assimilation and affecting photorespiration. Direct interaction of OsCV and stromal GS2 was confirmed *in vivo* (Sade et al., 2018).

In this work, we could confirm the role of CV2 in chloroplast degradation, by analyzing the effect of conditional overexpression of CV2-FLAG fusions on the accumulation levels of specific chloroplast proteins. Under overexpression conditions, the levels of a PSI protein (PsaB) and two PSII proteins (D1 and PsbO) were significantly reduced, confirming a functional conservation of soybean CV2. Unfortunately, we were not able to produce CV1-FLAG overexpressing lines. No CV1-FLAG protein was detected under inducible conditions in transgenic lines (T2) exhibiting high accumulation of transgene mRNA in the T1 generation, despite the use of a β-estradiol inducible promoter (data not shown). This result may indicate the occurrence of gene silencing in the CV1 overexpressing lines resulting from promoter leakage. In fact, soybean *CV1* transcript sequence contains a potential target for Arabidopsis miR419 that may explain the lack of protein accumulation in transgenic plants.

A search for *CV* orthologs and phylogenetic analysis in a number of plant species representative of the major plant families, revealed that CVs are only present in the genome of angiosperms, and that these genes exist since the earliest diverging angiosperm *Amborella trichopoda*. No *CV* orthologs were found in the genomes of gymnosperms or in any other tracheophyte or non vascular plant (**Table S2**). This indicates that the CVV pathway is involved in biological functions that are restricted to angiosperms, such as flower development, specific aspects of vascular development or N-recycling mechanisms. Taking into account the developmental regulation of expression of *CV* genes, the CVV pathway may be a specific angiosperm pathway involved in dPCD.

Interestingly, except for *Cephalotus follicularis* (Oxalidales) and *Genlisea aurea* (Lamiales) species, most carnivorous plants from different orders including Poales (Paepalanthus, Catopsis, Brocchinia), Oxalidales (Cephalotus), Caryophyllales (Drosera, Aldrovanda, Dionaea, Nepenthes, Drosophyllum, Triphyophyllum), Ericales (Roridula, Darlingtonia, Heliamphora, Sarracenia), and Lamiales (Philcoxia, Byblis, Ibicella, Proboscidea, Pinguicula, Genlisea, Utricularia), lack *CV* orthologs.

Despite their independent origins, carnivorous plants have convergently experienced a massive reduction in gene content during evolution, including the loss of most genes involved in non-carnivorous nutrition (Palfalvi et al., 2020). Carnivorous plants fix C from photosynthesis but absorb N and phosphorus from animal prey, helping them to survive and compete successfully in low-nutrient environments (Lin et al., 2021). To capture their pray, carnivorous plants use modified leaves called traps, which are associated with the loss of photosynthetically active tissue (Böhm and Scherzer, 2021). Interestingly, whereas most carnivorous plants employ their leaf-derived traps for both photosynthesis and prey capture (Freundm et al., 2022), the only two carnivorous that retained *CV* genes in their genome (Cephalotus and Genlisea) have conventional foliar leaves in addition to the specialized trap leaves. The fact that CV is only found in carnivorous species that maintain true leaves with high photosynthetic activity is consistent with the role that CV plays in the chloroplasts, where it is involved in the massive mobilization of N-rich thylakoidal and stroma proteins (via CVVs) from senescence tissue (source) to the developing sinks. In the case of many carnivorous plants, nutrients derived from pray digestion have been shown to increase sexual reproduction (Ellison and Gotelli, 2009), suggesting that chloroplast degradation may not be relevant for providing N for plant reproduction, eventually reducing the selective pressure on *CV* genes.

Several attempts to produce plants with delayed senescence have been carried out by interfering with proteins related to autophagy, SAVs, or ATI-PS, in most cases with unsatisfying results. Accelerated senescence has been observed in several autophagy-deficient mutants (Doelling et al., 2002; Hanaoka et al., 2002; Thompson et al., 2005; Phillips et al., 2008; Li et al., 2015). In the same direction, Arabidopsis ATI-KD mutants (in which ATI-1sequence was disrupted and ATI-2 was silenced; Honig et al., 2012) exhibited accelerated senescence under salt stress conditions (Michaeli et al., 2014). Despite being the main protease accumulated in the senescent leaves of *A. thaliana* and *Brassica napus* (Desclos et al., 2008; Poret et al., 2016), it has been shown that *SAG12* knockout mutants, failed to exhibit an altered senescence phenotype (James et al., 2018). However, these mutants showed lower yield and N content in their seeds when they were grown under N restriction (James et al., 2019). In contrast, Arabidopsis and rice *CV* gene silencing (Wang and Blumwald, 2014; Sade et al., 2018), and more recently, tomato *CV* gene disruption (Ahouvi et al., 2022), have demonstrated that interfering with CVV pathway is a feasible strategy to delay stress-induced senescence with minor or nor negative effects on crop productivity. In the case of soybean, *CV1* stands out as an interesting target for gene editing approaches for increasing tolerance to drought stress without compromising natural senescence, which involves *CV2*. The presence of two paralogous specialized genes in soybean minimizes possible yield penalties that may derive from editing target genes with relevant roles in developmental N mobilization processes.

## Data availability statement

The original contributions presented in the study are included in the article/**Supplementary Material**. Further inquiries can be directed to the corresponding author.

## Author contributions

SV contributed to conception of the study. AF and SV performed the experimental design in this study. AF and AC performed the experimental procedures. AF wrote the first draft of the manuscript. SV and EB critically revised the manuscript. All authors contributed to the article and approved the submitted version.
