# Supplementary material for: Functional specialization of chloroplast vesiculation (CV) duplicated genes from soybean shows partial overlapping roles during stress-induced or natural senescence
Source: Front Plant Sci. 2023 Jun 6;14:1184020. doi: 10.3389/fpls.2023.1184020 (PMC10280078; doi:10.3389/fpls.2023.1184020)
Supplement: Supplementary file 1 [file DataSheet_1.pdf]

Supplementary Material

Functional specialization of chloroplast vesiculation (CV) duplicated genes from soybean shows partial overlapping roles during stress-induced or natural senescence

Fleitas AL, Castro A, Blumwald E, Vidal S*.

*** Correspondence:** Sabina Vidal: svidal@fcien.edu.uy

Figure S1. Gene expression data of soybean genes encoding representative members of CCV, autophagy, SAVs and ATI-PS pathways.

Heat maps from publicly available gene expression data were created using Genevestigator (Hruz, et al., 2008). The data includes experiments in which soybean plants were exposed to dehydration or treated with NaCl.

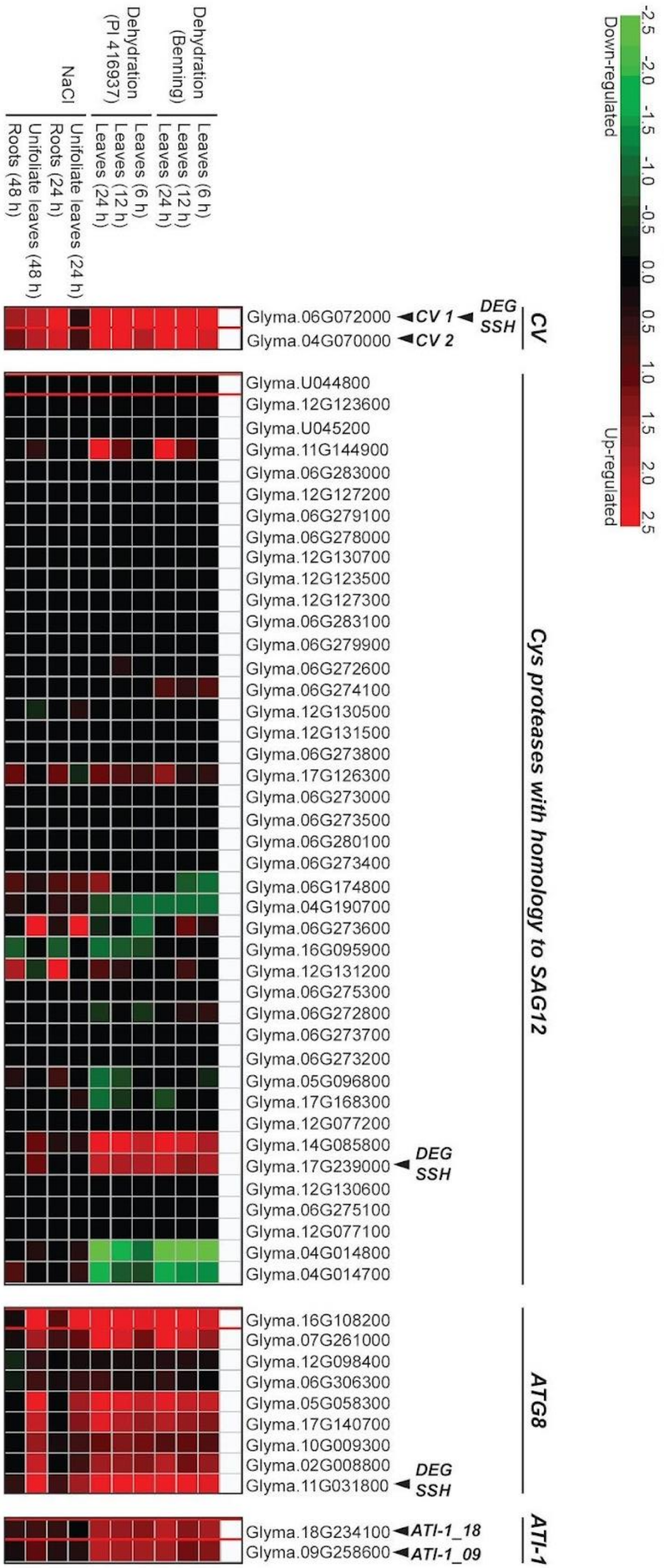

Figure S2. GUS activity in transgenic Arabidopsis plants in response to IAA (overnight treatments).

Ten-days old transgenic seedlings containing ProCV1-GUS or ProCV2-GUS gene constructs were treated with 100 nM IAA and GUS activity was detected by histochemical staining.

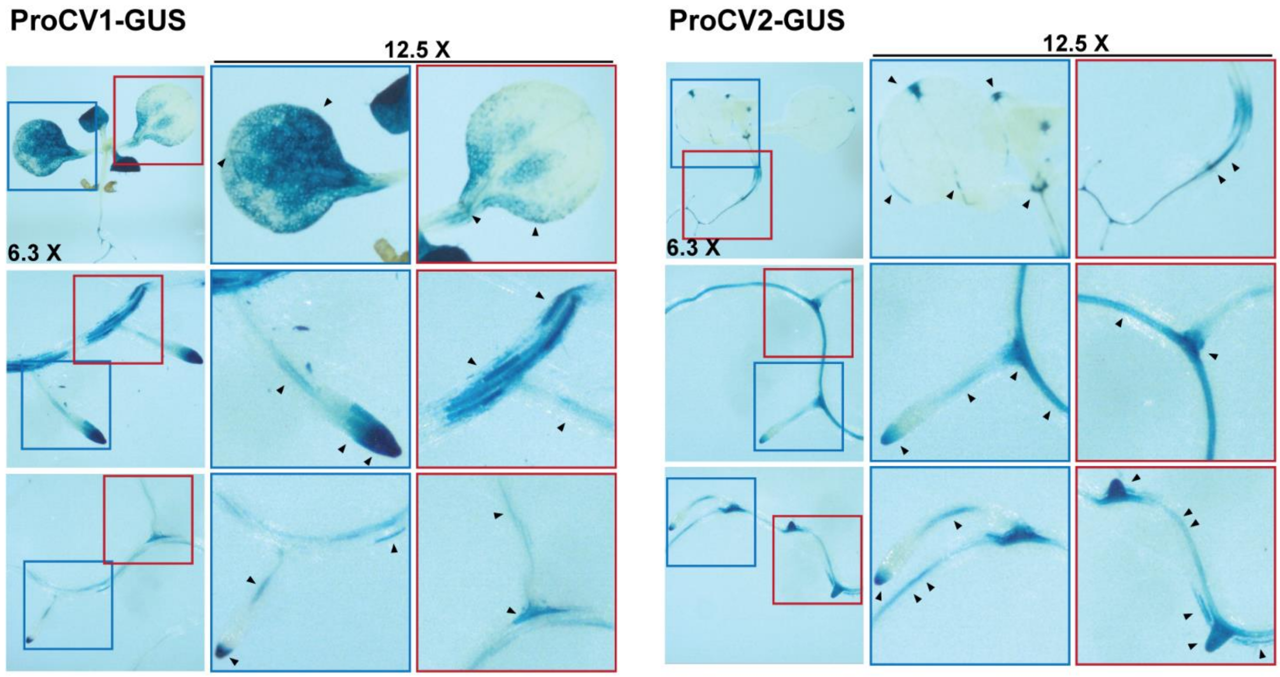

.

Table S1. List of primers employed in RT-qPCR experiments.

Gene	Primer name	Sequence 5'-3'
CV1	F-Gly.06G072000	TCAAACCAACCTTCCATTCC
	R-Gly.06G072000	CATTGCCACTGAATTGCACA
CV2	F-Gly.04G070000	CCAAATGGAGCCAGAAAAGA
	R-Gly.04G070000	ACAGTCTTGGTGGAGGAACG
ATG8j	F-Gly.11G031800	ATGTGATCCGCAAAAGGATT
	R-Gly.11G031800	AGAAACCCATCCTCGTCCTT
SAG12	F-Gly.17G239000	TGTCCTCTTGAGGGTGCTTC
	R-Gly.17G239000	GCCTTAATGCCTTCACTCCA
ATI1	F-Gly.09G258600	GCTCCTTCGCAGATCATTTC
	R-Gly.09G258600	GGAGCAGCTGCATATGTTGA
RR9	F-Gly.11G155100	AGCCTCCTCCCTGTATTGC
	R-Gly.11G155100	CCTTGATCTTTCTCAGCAG
ELF1B	F-Gly.02G276600	GTTGAAAAGCCAGGGGACA
	R-Gly.02G276600	TCTTACCCCTTGAGCGTGG

Table S2. Number of *CV* genes present in genomes of different plant species.

Mpo: Marchantia polymorpha, Ppa: Pyscomitrium pattens, Sfa: Sphagnum fallax, Smo: Selaginella moellendorffii, Pta: Pinus taeda, Pbr: Pinus brutia, Pha: Pinus halepensis subsp Brutia, Pta: Pinus tabulaeformis, Pla: Pinus laricio Savi, Pma: Pinus marítima, Pra: Pinus radiate, Gmo: Gnetum montanum, Gpa:Gnetum parvifolium, Ggn: Gnetum gnemon, Gha: Gnetum haianense, Gpe: Gnetum pendulum, Glu: Gnetum luofuense, Gma: Gnetum macrostachium, Gaf: Gnetum africanum, Gla: Gnetum latifolium, Ggn: Gnetum gneumonoides, Gul: Gnetum ula, Cmi: Cycas micholitzii, Gbi: Ginkgo biloba, Bdi: Brachypodium distachyon, Osa: Oryza sativa, Pha: Panicum hallii; Pvi: Panicum virgatum, Sit: Setaria italica, Svi: Setaria viridis, Sbi: Sorgum bicolor, Zma: Zea mays, Sly: Solanum lycopersicum, Kfe: Kalanchoe fedtschenkoi, Kla: Kalanchoe laxiflora, Egr: Eucalyptus grandis, Lus: Linum usitatissimum, Mes: Manihot esculenta, Ptr: Populus trichocarpa, Csi: Citrus sinensis, Ath: Arabidopsis thaliana, Csa: Cucumis sativus, Fve: Fragaria vesca; Gma: Glycine max, Mdo: Malus domestica, Mtr: Medicag truncatula, Pvu: Phaseolus vulgaris, Ppe: Prunus persica, Tpr: Trifolium pratense, Cre: Chlamydomonas reinhardtii.

Node	Cre	*Mpo*	*Ppa*	*Sfa*	*Smo*
**Chlorophyte**	-				
**Embriophyte**		-	-	-	
**Trachophyte**					-

Node	*P<br>ab*	*P<br>gl*	*P<br>si*	*P<br>pi*	*P<br>sy*	*P<br>ta*	*P<br>br*	*P<br>ha*	*P<br>ta*	*P<br>la*	*P<br>ma*	*P<br>ra*	*G<br>mo*	*G<br>pa*	*G<br>gn*	*G<br>ha*	*G<br>pe*	*G<br>lu*	*G<br>ma*	*G<br>af*	*G<br>la*	*G<br>gn*	*G<br>ul*	*C<br>mi*	*G<br>bi*
**Embriophyte**																									
**Trachophyte**																									
**Gymnosperm**																									
**Pinaceae**	-	-	-	-	-	-	-	-	-	-	-	-													
**Gnetidae**													-	-	-	-	-	-	-	-	-	-	-		
**Cycadidae**																								-	
**Ginkgoidae**																									-

Table S2, continued.

Node	*Bdi*	*Osa*	*Pha*	*Pvi*	*Sit*	*Svi*	*Sbi*	*Zma*	*Sly*	*Kfe*	*Kla*	*Egr*	*Lus*	*Mes*	*Ptr*	*Csi*	*Ath*
**Angiosperm**																	
**Grass**	1	1															
**Panicoideae**			2	4	2	2	2	2									
**Eudicot**										1	2						
**Pentapetalae**																	
**Asterid**									1								
**Rosid**												1					
**Malvidae**																	
**Malpighiales**													2	2	1		
**Citrus**																1	
**Brassicaceae**																	1

Node	*Csa*	*Fve*	*Gma*	*Mdo*	*Mtr*	*Pvu*	*Ppe*	*Tpr*
**Fabidae**	1	1	2	2	1	1	1	2
**Chlorophyte**								

Table S3. Transcriptional response of soybean genes encoding representative members of CCV, autophagy, SAVs and ATI-PS pathways according to publicly available transcriptomic data.

DS: Drought Sensitive, DT: Drought Tolerant, SW: Slow wilting. UP: Up regulated, DW: Down regulated, C: Constant, ND: Not determined, LE: Low expression. Experimental conditions: Genevestigator: 6, 12 and 24 h dehydration treatment; Shin et al., 2015: R2 plants, 6, 12 and 24 h air dry treatment; Ha et al., 2015: V2 plants, 2 and 10 h air dry treatment, plant relative water content was 18% for W82 and 40% for DT2008; Le et al., 2012: V6 to R2 plants, 6 d water withholding, soil water content < 5%, leaf relative water content 60% of well-watered plants; Gallino et al., 2018: V5 plants, water withholding to 50% or 25% soil water content.

Gene	Locus name	Regulation				
**Source**		Genevestigator	Shin et al., 2015	Ha et al., 2015	Le et al., 2012	Gallino et al., 2018
**Genotype**		Benning (DS), PI416937 (SW)	Benning (DS), PI416937 (SW),	DT2008 (DT), Williams (DS)	Williams82	TJS2049 (DS), N7001 (SW)
**Tissue**		Leaf	Leaf	Root	Leaf	Leaf
**Method**			Ilumina seq	Affimetrix 66K	Affimetrix 66K	SSH
**CV1**	Glyma.06G072000	UP	UP	UP	UP	UP
**CV2**	Glyma.04G070000	UP	UP	C	UP	ND
**ATG8**	Glyma.11G031800	UP	UP	C	UP	UP
	Glyma.15G108200	UP	UP	C	UP	ND
	Glyma.07G261000	UP	UP	C	UP	ND
	Glyma.02G008800	UP	UP	C	UP	ND
	Glyma.10G009300	UP	UP	C	UP	ND
	Glyma.05G058300	UP	UP	C	UP	ND
	Glyma.17G140700	UP	UP	C	UP	ND
	Glyma.12G098400	C	C	C	DW	ND
	Glyma.06G306300	C	C	C	DW	ND
		.				
**SAG12**	Glyma.14G085800	UP	UP	UP	C	UP

Glyma.17G239000	UP	UP	UP	C	UP
Glyma.11g144900	UP	C	C	UP	ND
Glyma.17G126300	UP	LE, C	C	UP	ND
Glyma.06G274100	UP	ND	C	UP	ND
Glyma.12g131200	UP	ND	C	UP	ND
Glyma.16G095900	DW	LE, C	C	DW	ND
Glyma.17G168300	DW	LE, C	C	UP	ND
Glyma.04G190700	DW	LE, C	C	DW	ND
Glyma.06G174800	DW	C	C	DW	ND
Glyma.04G014800	DW	LE,C	C	DW	ND
Glyma.06G273600	UP(DS)/DW(DT)	LE, C	C	DW	ND
Glyma.05g096800	C	ND	C	DW	ND
Glyma.06G272600	C	ND	C	DW	ND
Glyma.06g272800	C	ND	C	DW	ND
Glyma.06g273000	C	ND	C	DW	ND
Glyma.06g273200	C	ND	C	DW	ND
Glyma.06g273400	C	ND	C	DW	ND
Glyma.06g273500	C	ND	C	DW	ND
Glyma.06G273700	C	ND	C	UP	ND
Glyma.06g273800	C	ND	C	UP	ND
Glyma.06g275100	C	ND	C	DW	ND
Glyma.06g275300	C	ND	C	DW	ND
Glyma.06G278000	C	ND	C	DW	ND
Glyma.06G279100	C	ND	C	DW	ND
Glyma.06g280100	C	ND	C	DW	ND
Glyma.06G283000	C	ND	C	DW	ND
Glyma.06G283100	C	ND	C	UP	ND
Glyma.12G077100	C	ND	C	DW	ND
Glyma.12G077200	C	ND	C	DW	ND
Glyma.12g123500	C	ND	C	UP	ND
Glyma.12g123600	C	ND	C	DW	ND
Glyma.12g127200	C	ND	C	UP	ND
Glyma.12g127300	C	ND	C	DW	ND
Glyma.12g130500	C	ND	C	DW	ND
Glyma.12G130600	C	ND	C	DW	ND
Glyma.12g130700	C	ND	C	DW	ND
Glyma.12g131500	C	ND	C	DW	ND

	Glyma.16g095900	DW	ND	ND	ND	ND
	Glyma.U044800	C	ND	ND	ND	ND
	Glyma.U045200	C	ND	ND	ND	ND
**ATI1**	Glyma.18G234100	UP	UP	C	DW	ND
	Glyma.09G258600	UP	UP	C	DW	ND
